# Ammonia as a parameter shaping habitability on icy moons

**DOI:** 10.1093/femsmc/xtag015

**Published:** 2026-03-26

**Authors:** Cassie M Hopton, Charles S Cockell

**Affiliations:** UK Centre for Astrobiology, School of Physics and Astronomy, University of Edinburgh, James Clerk Maxwell Building, Peter Guthrie Tait Road, Edinburgh EH9 3FD, United Kingdom; UK Centre for Astrobiology, School of Physics and Astronomy, University of Edinburgh, James Clerk Maxwell Building, Peter Guthrie Tait Road, Edinburgh EH9 3FD, United Kingdom

**Keywords:** astrobiology, icy moons, Enceladus, Titan, ammonia, ocean worlds

## Abstract

The search for life now extends beyond the traditional habitable zone to include the icy moons of Jupiter and Saturn. These moons feature ice-covered surfaces overlying substantial oceans formed primarily of liquid water and other potential constituents, such as ammonia. On Earth, ammonia supports biochemistry at low concentrations by providing nitrogen but becomes disruptive at higher concentrations. Ammonia could therefore influence the habitability of extraterrestrial oceans, yet this topic has received limited attention in the literature. This review synthesises current research on ammonia in Saturn’s icy moons, Enceladus and Titan, and its effects on terrestrial life. We summarize the celestial incorporation, speciation, and phase behaviour of ammonia and review data on its occurrence and concentration in icy moon oceans. We examine the role of ammonia in prebiotic chemistry, biochemistry, and toxicity. Focusing on bacteria, we compare known survival limits in ammonia to estimated ammonia concentrations on Enceladus and Titan. We find that bacterial survival limits exceed concentrations estimated on Enceladus, but are below those estimated on Titan, and propose that ammonia measurements are crucial for assessing extraterrestrial habitability. Finally, we highlight outstanding knowledge gaps and challenges that influence our understanding of how ammonia shapes the potential for life beyond Earth.

## Teaser

This review explores how ammonia in the subsurface oceans of Saturn’s icy moons, Enceladus and Titan, could shape conditions for life and influence their potential to host microbes.

## Introduction

A habitable environment can be defined as “an environment capable of supporting the activity of at least one known organism” (Cockell et al. 2016). The question of habitability beyond Earth has been the subject of both philosophical and scientific interest for two millennia. As early as the 6^th^ century BCE, Greek philosopher Anaximander speculated about multiple worlds that may harbour extraterrestrial life, and a water origin of human life on Earth (Rovelli 2023). This early curiosity evolved with scientific discovery. In the 17^th^ century, four moons of Jupiter (Io, Europa, Ganymede, and Callisto) were discovered by Galileo and subsequently dubbed the “Galilean moons” (Soderblom 1980), a discovery that expanded the known celestial bodies in the Solar System. By the 20^th^ century, the role of water in the emergence of life became evident. Stanley Miller and Harold Urey demonstrated that amino acids could form from a mixture of ammonia, methane, hydrogen, and water (Miller 1955). This experiment provided evidence for Oparin’s theory of abiogenesis, a “primordial soup” origin of life hypothesis whereby a mixture of inorganic compounds in water gave rise to living organisms (Oparin 1938).

Almost two decades later, the first physical evidence of extraterrestrial water came from water-bearing minerals detected in lunar samples returned by Apollo 11 (Goles 1971, Levinson and Taylor 1971). Building on this discovery, the Galileo mission subsequently provided data to support a subsurface ocean of liquid water below the thick ice crust of one of the Galilean moons, Europa (Reynolds et al. 1983, Anderson et al. 1998, Greeley et al. 1998). At the turn of the 21^st^ century, scientific attention to extraterrestrial water continued, and NASA introduced a new approach as part of the Mars Exploration Program: “Follow the water” (Hubbard et al. 2002). The approach was designed initially to discover records of biological processes on Mars. However, in 2005, the Cassini–Huygens mission revealed plumes of water vapour and ice erupting from Saturn’s moon Enceladus (Hunter et al. 2006). Now, strong evidence for subsurface oceans of liquid water encased below surface ice is established not only on Europa and Enceladus (Roberts and Nimmo 2008, Tobie et al. 2008, Miles et al. 2025), but also Ganymede (Showman et al. 2004, Saur et al. 2015), Callisto (Zimmer et al. 2000, Cochrane et al. 2025), Neptune’s moon Triton (Gaeman et al. 2012), and the Dwarf planet Ceres, amongst others (McCord and Sotin 2005). Saturn’s largest moon, Titan, could also harbour a subsurface liquid ocean (Gabriel et al. 2005, Baland et al. 2011, Bills and Nimmo 2011) or a high-pressure ice layer containing pockets of partial melt (Petricca et al. 2025). With these discoveries, the “follow the water” approach now applies not only to Mars but also to the icy moons of Jupiter, Saturn, and other ocean-bearing planetary bodies across the universe.

Beyond the frost line, far from the Sun’s warming influence, the preservation of a liquid layer within icy moons and other celestial bodies could appear paradoxical. Yet, such layers are possible through tidal or radiogenic heating, or the presence of freezing point depressants such as salt or ammonia. Indeed, temperatures are low enough beyond the frost line for volatile compounds like ammonia to condense into solid ice and accrete with planetary bodies. As such, ammonia has been detected in the plumes of Enceladus by Cassini (Waite et al. 2009) and on the surface of Ceres (King et al. 1992). When considering astrobiology, the presence of such a constituent within the oceans of extraterrestrial bodies would be significant. On Earth, ammonia has been implicated in facilitating prebiotic chemistry as a nitrogen source (Wigley and Brimblecombe 1981, Martin et al. 2008, Sojo et al. 2016, Nishizawa et al. 2021), and in modern systems, plays an integral role in the global nitrogen cycle (Fowler et al. 2013) and the synthesis of nucleic acids and amino acids (Adeniyi et al. 2023, Fu et al. 2023). However, high concentrations of ammonia are also known to be toxic across all the domains of life (Eno et al. 1955, Thurston et al. 1981, Leejeerajumnean et al. 2000, Hachiya et al. 2021). On Earth, what constitutes “high” concentrations of ammonia is not well defined, but could be considered those that exceed 6 ppm (≈ 0.00035 M) in waters and soils (Roney et al. 2004). Ammonia could therefore be a prominent factor influencing habitability as we know it if present within icy moon subsurface oceans above this level.

Ammonia can exist in two molecular forms: a weak base, ammonia (NH_3_), and a weak acid, the ammonium ion (NH_4_^+^). Henceforth, the term “total ammonia” refers collectively to the concentration of both NH_3_ and NH_4_^+^ in an aqueous environment. The position of equilibrium between these two species is dictated primarily by pH; under high pH, NH_3_ dominates, and under low pH, NH_4_^+^ dominates. While NH_4_^+^ can disrupt the ionic balance of organisms, the permeation of this species is regulated by membrane transporters (Moser 1987, Wacker et al. 2014), and the tolerance of organisms to NH_4_^+^ is high (i.e., up to and possibly beyond 2 M). For these reasons, NH_4_^+^ is characterized as “non-toxic”. NH_3_ is a proton (H^+^) acceptor, and has properties (e.g., small size, uncharged) which facilitate passive permeation through biological membranes. As a result, NH_3_ readily reacts with H^+^ to raise both extracellular and intracellular pH, disrupt the proton motive force, and form NH_4_^+^ intracellularly (Rose et al. 2005, Bosoi and Rose 2009, Angelova et al. 2022). High concentrations of NH_4_^+^ delivered through NH_3_ permeation can disrupt membrane potential, ionic balance, and metabolism (Wang et al. 2018, Xiao et al. 2019, Shi et al. 2020). NH_3_ is therefore considered toxic and has the potential to significantly disrupt the physicochemical environment as well as the biochemistry within living organisms. To date, 0.5 M is the highest concentration at which a bacterium has been observed to survive in NH_3_ (Leejeerajumnean et al. 2000).

Given these effects, the occurrence of NH_3_ may thus limit the habitability potential of extraterrestrial oceans. Focusing on icy moons, it is not yet known if all of these oceans contain significant amounts of NH_3_, if any. Owing to the thermal conditions of the Jovian nebula and current geochemical models of Europa’s ocean, it is unlikely that NH_3_ predominates there. Ganymede and Callisto may harbour subsurface waters containing NH_3_, but available compositional constraints remain insufficient to draw firm conclusions. The ocean of Enceladus is estimated to have a pH of ∼10.6 (Glein and Truong 2025), and Titan’s ocean may be of a similarly high pH, or greater (Marion et al. 2012, Brassé et al. 2017, Leitner and Lunine 2019). Both oceans are expected to contain dissolved NH_3_ and/or NH_4_^+^ based on current interior and thermal evolution models. For these reasons, this review focuses on Enceladus and Titan, where well-constrained formation and physicochemical conditions are most conducive to elevated NH_3_ abundances.

Despite the possibility of NH_3_ in the oceans, there are several properties that underpin Enceladus and Titan as prominent targets in the search for life beyond Earth. First, in addition to liquid water, there is evidence of tidal heating (Carr et al. 1998, Nimmo et al. 2007, Roberts and Nimmo 2008, Chen et al. 2014, Běhounková et al. 2021) or radiogenic heating (Grasset et al. 2000, Sohl et al. 2014) within both icy moons. Hydrothermal chemistries at the ocean floor of Enceladus are conceivable (Hand et al. 2007, Matson et al. 2007, Vance et al. 2007, Zolotov Mikhail Y. 2007, Hsu et al. 2015), akin to the origin-of-life hydrothermal vents on Earth (Hand et al. 2007, Matson et al. 2007, Vance et al. 2007, Zolotov Mikhail 2007). Redox chemistry is plausible on Titan (McKay and Smith 2005, McKay 2016). Additionally, all the essential elements for life on Earth, the CHNOPS suite (carbon, hydrogen, nitrogen, oxygen, phosphorus, sulphur), have been detected or are otherwise feasible on Enceladus (Waite et al. 2009, Waite et al. 2017, Postberg et al. 2023, Xu et al. 2025). Many of the CHNOPS have also been detected on Titan (Sagan et al. 1992, Hiscox 2000, Owen 2000, Nixon 2024). Organics have been directly detected in the plume of Enceladus (Waite et al. 2009, Postberg et al. 2018, Khawaja et al. 2025), and in the atmosphere of Titan (Lellouch et al. 1989, Niemann et al. 2005). Pressures in the ocean of Enceladus could range from 1 to 30 MPa (Vance et al. 2018) and could reach 800 MPa on Titan at the seafloor (Journaux et al. 2020). While this may limit habitability prospects to piezotolerant and piezophilic organisms, it is the combination of available heat, energy, nutrients, and organics, in addition to liquid water, that makes these icy moons compelling targets in astrobiology (Fortes 2000, McKay et al. 2008).

The potential for habitability on Enceladus and Titan has been widely speculated given the favourable internal conditions presented. However, even with its potential impact on life, the significance of NH_3_ is little discussed or represented in astrobiology literature. This interdisciplinary review synthesises current research on NH_3_ in the high pH oceans of Saturn’s icy moons, Enceladus and Titan, and examines its effects on terrestrial life. We discuss the geochemical occurrence, chemical behaviour, and biological impacts of total ammonia on Earth, drawing implications for the habitability of these extraterrestrial, ammoniacal oceans.

## Origin, speciation, and phase behaviour of ammonia

NH_3_ is a primordial molecule. It is found in celestial bodies across the universe, including stars (Schmidt et al. 2011, Wong et al. 2018), planets (Henderson-Sellers and Schwartz 1980, Cleland and Rimmer 2022, Moeckel et al. 2023, Irwin et al. 2025), moons (Nelson et al. 2009, Waite et al. 2009, Holler et al. 2017), comets (Wyckoff et al. 1989, Feldman et al. 1993, Poch et al. 2020), asteroids (Pizzarello et al. 2011, Glavin et al. 2025), and distant galaxies (Ao et al. 2011, Mills and Morris 2013), as well as the interstellar medium (Cheung et al. 1968, Doherty et al. 2022).

In the near vacuum pressures of space, NH_3_ is found primarily as solid ice. Temperatures are below the sublimation point (−73°C) (Glasser 2009) and NH_3_ ice can be trapped during accretion of cold bodies, including icy moons. Within icy moons, subsequent internal heating can later release NH_3_ as a gas as subsurface oceans form (Spohn and Schubert 2003, Tobie et al. 2012, Vance et al. 2018). NH_3_ gas readily dissolves in water (Hales and Drewes 1979, Dasgupta and Dong 1986, Simonelli et al. 1998). The resulting ammoniacal waters, a mixture of NH_3_ and water, can have a freezing point as low as −100°C; dissolution of NH_3_ in water interferes with water–water hydrogen bonding, thus acting to lower the freezing point. This antifreeze behaviour has led to the speculation that ammonia–water may maintain subsurface liquid oceans in icy moons, despite cold surface temperatures (Johnson and Nicol 1987, Croft et al. 1988, Leliwa-Kopystyński et al. 2002, Chua et al. 2023).

Within aqueous environments, NH_3_ undergoes a particular reaction. As a H^+^ acceptor, the dissolution of NH_3_ into water leads to protonation and the formation of NH_4_^+^ and hydroxide ions (OH^−^), according to the following equation:


\begin{eqnarray*}
N{{H}_3} + {{H}_2}O \rightleftharpoons NH_4^{\ + } + O{{H}^ - }
\end{eqnarray*}

**Figure 1:**
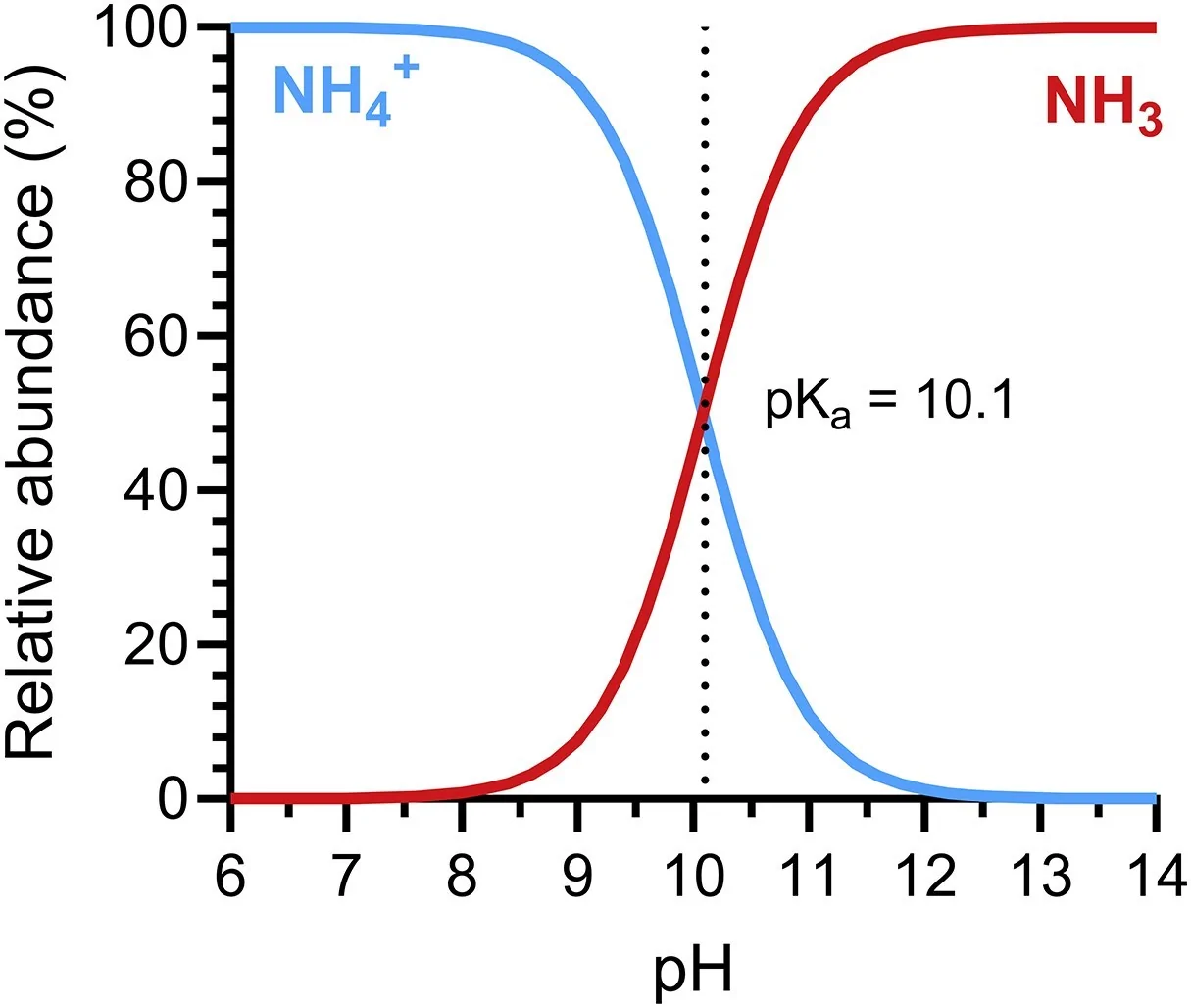
Abundance of NH_3_ and NH_4_^+^ as a function of pH. The pK_a_ of the $N{{H}_3} \rightleftharpoons NH_4^{\ + }$ system was calculated based upon an ionic strength of 0.3 M, a temperature of 0°C, and 0.1 MPa pressure. Pressure may vary, but these ionic strengths and temperatures are parameters expected within the ocean of Enceladus (Glein and Truong 2025). A pK_a_ of 10.1 was calculated, indicated by a dotted line from the *x*-axis.

The position of equilibrium between NH_3_ and NH_4_^+^ in water is dictated primarily by pH as described by the Henderson–Hasselbalch equation, where low pH promotes the formation of NH_4_^+^ and high pH promotes the formation of NH_3_. However, the speciation of total ammonia is also influenced by salinity, temperature, and pressure. Higher salinity, colder temperatures, and elevated pressures increase the negative logarithm of the acid dissociation constant (pK_a_) of the $N{{H}_3} \rightleftharpoons NH_4^{\ + }$ equilibrium, thus favouring the protonation of NH_3_ (forming NH_4_^+^). Lower salinity, warmer temperatures, and lower pressure exert the opposite effect (Neuhausen and Patrick 1921, Emerson et al. 1975, Hales and Drewes 1979, Dasgupta and Dong 1986). Thus, the speciation of total ammonia in icy moon interiors is dictated by these physicochemical parameters (Fig. 1).

**Figure 2:**
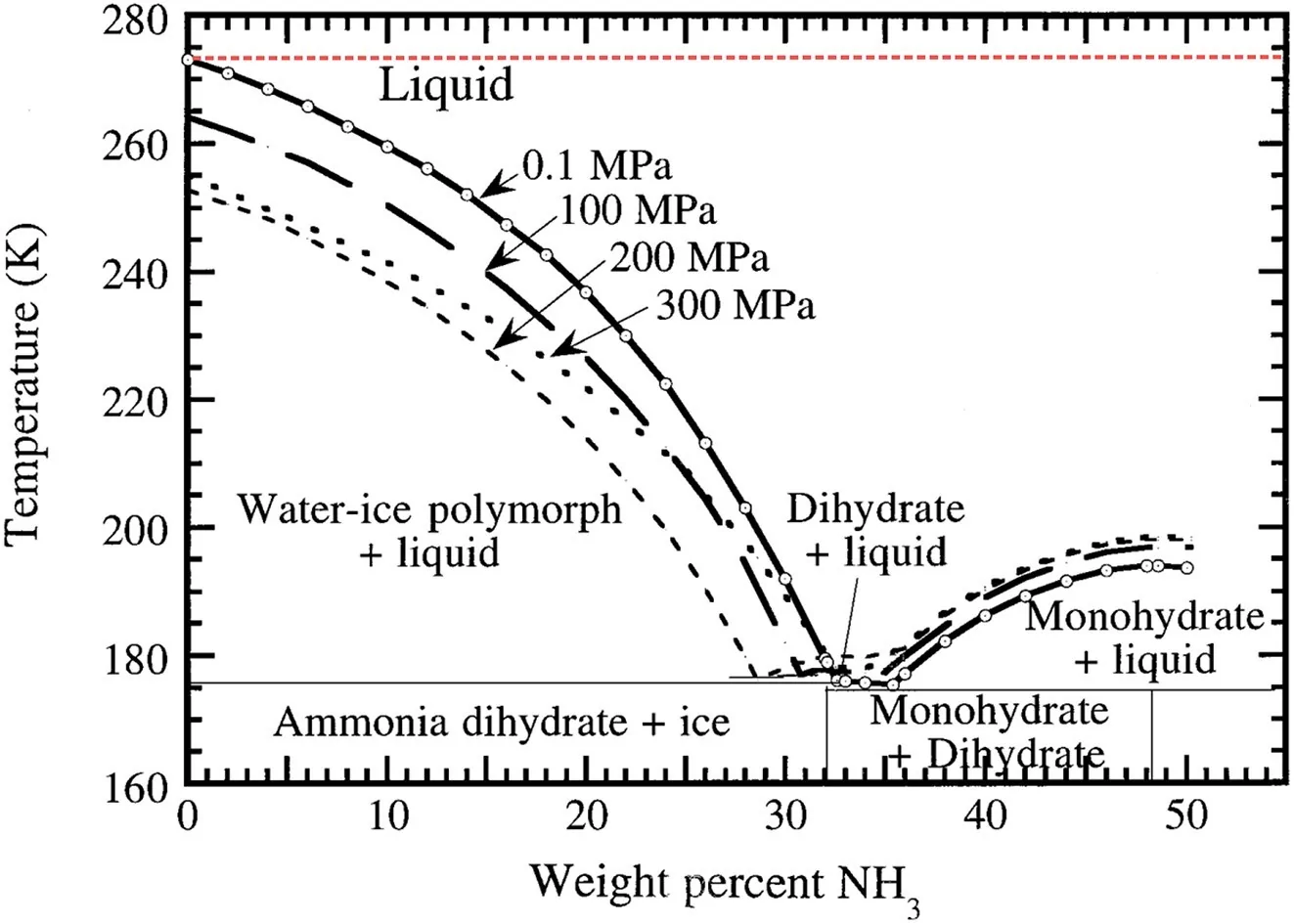
Phase diagram of ammonia–water mixtures. Phase behaviour against the concentration-temperature plane is depicted, showing the influence of pressures at 0.1 MPa, 100 MPa, 200 MPa, and 300 MPa. The ammonia–water solidus at 176.16 K is illustrated with a solid line. A dashed line (red) has been superimposed on the figure to highlight phase behaviour at a temperature of 273.15 K (0°C). Figure adapted from Hogenboom et al. (1997) with permission from Elsevier.

In addition to speciation, pressure can also alter the phase behaviour of NH_3_. On icy moons, gravitational forces acting on thick water layers, combined with the substantial thickness of ice shells in the order of several to hundreds of kilometres, generate hydrostatic pressures of tens to a few hundred MPa at the seafloor, and up to a few GPa at the seafloor of larger moons (Billings and Kattenhorn 2005, Nimmo and Bills 2010, Baland et al. 2014, Čadek et al. 2016, Lucchetti et al. 2017, Vance et al. 2018, Levin et al. 2026). In mixtures of ammonia–water at standard pressure, the boiling point can remain above 0°C and may only be depressed by a few degrees from the standard boiling point of water (100°C) when mixed at low concentrations of NH_3_, i.e., 1% NH_3_. However, elevated pressures can increase the boiling point, and also alter the freezing point (Clifford and Hunter 1933). For example, at 100 MPa pressure, the freezing point of ammonia–water with a concentration of 1% NH_3_ by weight is approximately −9°C, slightly colder than the freezing point at 0.1 MPa (−3°C) (Fig. 2). At 300 MPa, the freezing point temperature decreases substantially to −33°C, and so on (Leliwa-Kopystyński et al. 2002).

Current models estimate that the temperature at the base of the ice shell on icy moons ranges from −20 °C to near 0°C, although subsurface oceans could be warmer near hydrothermal regions or exhibit inverted temperature gradients (Kargel et al. 2000, Marion et al. 2003, Melosh et al. 2004, Matson et al. 2012, Sohl et al. 2014, Glein et al. 2015). Given these conditions, NH_3_ most likely exists in the liquid phase within icy moon oceans, with warmer regions exhibiting NH_3_ gas and some colder regions possibly exhibiting NH_3_ ice, depending on the temperature and pressure of the region. If permitted by sub-zero temperatures (e.g., −98°C), ammonia–water may also form solid, crystalline ammonia hydrate structures (Fig. 2) (Hogenboom et al. 1997, Muñoz-Iglesias and Prieto-Ballesteros 2021).

Given the toxicity of NH_3_, it is critical to understand whether icy moon conditions could permit this species to persist, and if so, the concentrations at which it may occur. For icy moons Enceladus and Titan, there is substantial data from which to extrapolate pressure, temperature, salinity, and pH. By integrating geochemical models, spacecraft observations, and laboratory analogues, we can begin to constrain the abundance and speciation of total ammonia, both of which are essential for assessing habitability.

## Enceladus: plumes of ammonia

**Figure 3:**
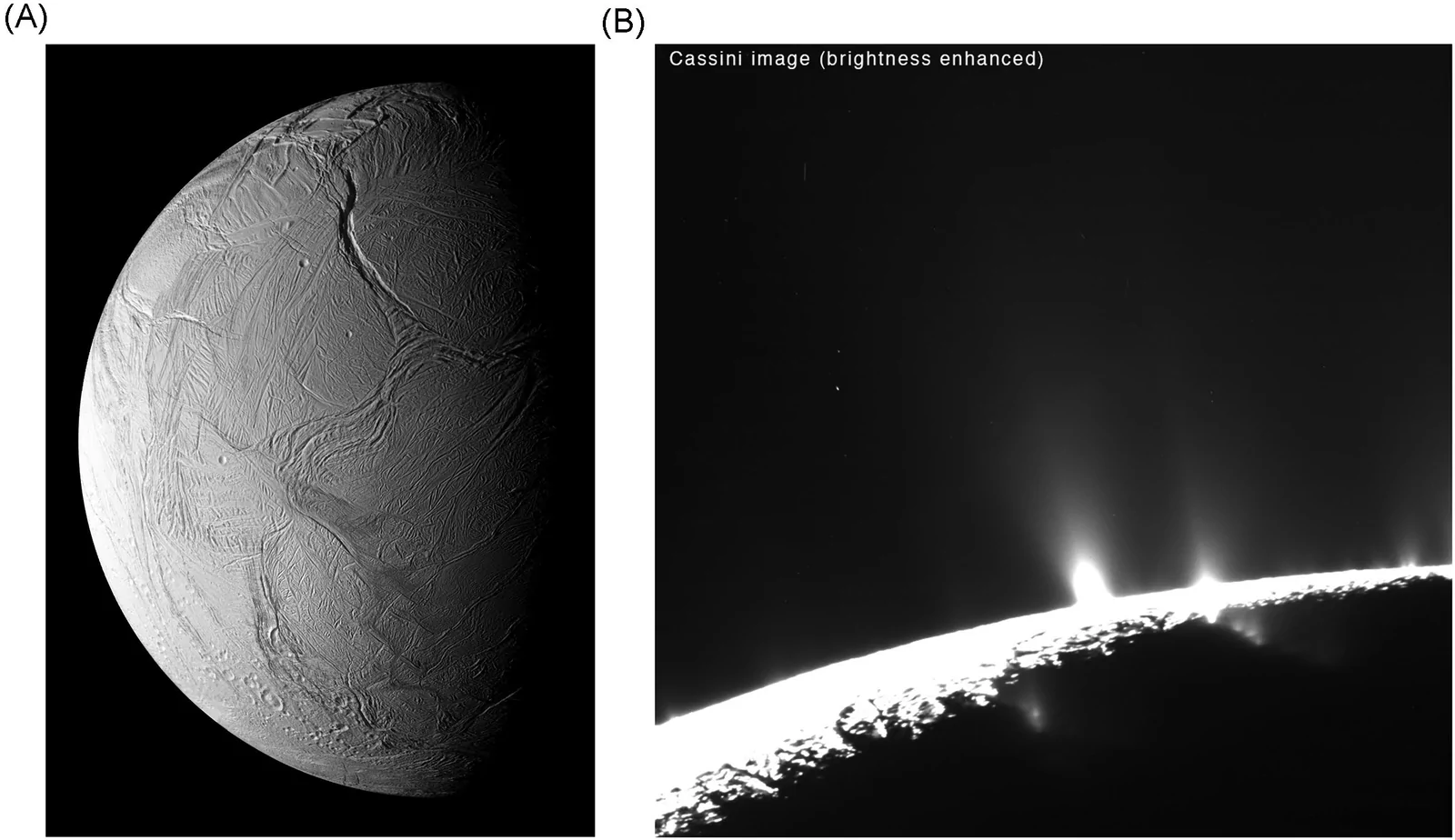
Aqueous NH_3_ within Enceladus. (A) Surface of Enceladus captured by the Cassini Imaging Science Subsystem. Portions of the tiger stripe fractures from which plumes of water emanate are visible, surrounded by a circumpolar belt of mountains. Image credits: NASA/JPL/Space Science Institute. (B) Discrete plumes expelled from Enceladus’ surface as captured by Cassini. These plumes were later confirmed to consist of water and other molecules such as NH_3_. Image credits: NASA/JPL-Caltech/SSI/PSI.

In the 1980s, investigations with Voyager 2 of the 6^th^ largest moon of Saturn, Enceladus, revealed a geologically young surface. Co-occurrence of the densest region of Saturn’s E ring with Enceladus' orbit indicated possible evidence of eruptive activity (Smith et al. 1982). Over two decades later, the revelations from Cassini were groundbreaking. Multiple flybys of Enceladus revealed jets of icy particles erupting from 130 km long fractures in the Southern pole, the “tiger stripes”, and confirmed deposition of particles into Saturn’s E ring (Fig. 3) (Porco et al. 2006, Spitale and Porco 2007). Analysis of the plumes by two mass spectrometers onboard Cassini, the Ion and Neutral Mass Spectrometer (INMS) and Cosmic Dust Analyzer (CDA), elucidated the presence of water vapour, ice particles, simple organics (e.g., benzene), complex macromolecular organics, and gases such as deuterium, hydrogen, carbon dioxide, carbon monoxide, methane, and NH_3_ (Waite et al. 2006, 2009, 2017, Postberg et al. 2018).

The discoveries made by the Cassini mission reshaped our view of Enceladus into a prime candidate for astrobiology and further exploration. In particular, the discovery of vapour and NH_3_ on Enceladus posed an enthralling question as to whether there could be liquid water beneath the ice crust. As aforementioned, ammoniacal solutions can remain liquid as low as −100°C, depending on the concentration of NH_3_ (Johnson and Nicol 1987, Croft et al. 1988, Leliwa-Kopystyński et al. 2002, Chua et al. 2023). The detection of NH_3_ by Cassini therefore provided strong indirect evidence for the existence of subsurface liquid water on Enceladus. Further evidence for a liquid subsurface reservoir with NH_3_ was gleaned by the presence of ^40^Ar in the plume (Waite et al. 2009). Originally in reference to Titan, Engel et al. (1994) suggested that a water ocean preserved in the liquid state by NH_3_ could dissolve sodium and potassium from silicate rock into fluid, leading to eventual decay of potassium-40 into ^40^Ar that could later surface into the atmosphere by cryovolcanism. The detection of ^40^Ar therefore provided further indications of an ocean containing NH_3_ with active chemical exchange with its rocky core. This inference was strengthened by the detection of sodium-rich salts, such as sodium chloride (NaCl) and sodium bicarbonate (NaHCO_3_), in Saturn’s E ring and freshly ejected plume particles; such salts can only arise from a liquid water origin (Postberg et al. 2009, 2011).

Cassini’s INMS measurements of Enceladus’ plumes showed NH_3_ mixing ratios between 0.4% and 1.3%. The observed variability is thought to reflect compositional differences among plume eruptions during multiple flybys (Waite et al. 2006, 2009, 2017). It is notable that the concentrations align well with NH_3_-to-water ratios observed in comets between 0.01% to 1.5% (Wyckoff et al. 1989, Meier et al. 1994, Palmer et al. 1996), supporting the notion of primordial incorporation of solid NH_3_ into Enceladus during its accretion. Mass spectra from the CDA subsequently indicated spectral features consistent with NH_4_^+^ in the ejected ice grains, further supporting the hypothesis of a subsurface ocean with dissolved total NH_3_ (Khawaja et al. 2019). Incorporating the Cassini NH_3_ data, a bulk molecular abundance of total ammonia in the Enceladus ocean between 0.011%–0.169% has been derived, approximating to an oceanic concentration between ≈ 0.01–0.1 M. This corresponds to 0.001%–0.006% NH_3_ and 0.01%–0.163% NH_4_^+^ in an ocean between pH 7.95 and 9.05 (Fifer et al. 2022). Fifer et al. (2022) reasoned that the NH_3_ plume composition does not directly reflect oceanic NH_3_ concentration; volatile gases such as NH_3_ likely undergo exsolution from the liquid phase during plume formation, depleting NH_3_ concentrations in the plume compared to the ocean.

However, the estimations of total ammonia in the ocean of Enceladus are evolving with new data. Phosphate-rich grains have been discovered in the plume ejecta, with species of Na_2_HPO_4_ and Na_3_PO_4_ reproducing the peak pattern of the CDA spectra most accurately (Postberg et al. 2023). Phosphorus in ionic forms of HPO_4_^2−^ and PO_4_^3−^ occur at high pH values between pH 7 and pH 12, and greater than pH 12, respectively. Thus, the presence of these phosphate species constrains the ocean from pH 10.1 to pH 11.6 according to recent geochemical modelling (Glein and Truong 2025). Reconstruction of the ocean chemistry place concentrations of NH_3_ at 0.0181 molal (≈ 0.0181 M) (Glein and Truong 2025). These concentrations are low compared to bulk composition. Thermodynamic modelling of ammonia–water mixtures indicates such concentrations would not be sufficient to sustain liquid water if the ocean temperature were found to be below zero (Croft et al. 1988, Kargel 1992, Hogenboom et al. 1997).

The ocean of Enceladus is thought to be near 0°C at the ice-ocean interface (Matson et al. 2012, Glein et al. 2015). At near 0°C, the pK_a_ for the reaction $N{{H}_3} + {{H}_2}O \rightleftharpoons NH_4^{\ + } + O{{H}^ - }$ is ∼10.1 (Bates and Pinching 1949). Thus, as per the Henderson–Hasselbalch equation and current pH estimations, the ocean would bear NH_3_ predominantly, as per observations from Cassini (Glein and Truong 2025). However, at the concentrations estimated, it is unlikely that NH_3_ contributes significantly to the maintenance of liquid water. Although NH_3_ may facilitate the liquid state in colder regions of the ocean where the temperature may fall slightly below 0°C, internal heat generated by tidal heating is thought to be the primary driver of liquid state preservation (Nimmo et al. 2007, Roberts and Nimmo 2008, Chen et al. 2014).

## An ammonia reservoir on titan

**Figure 4:**
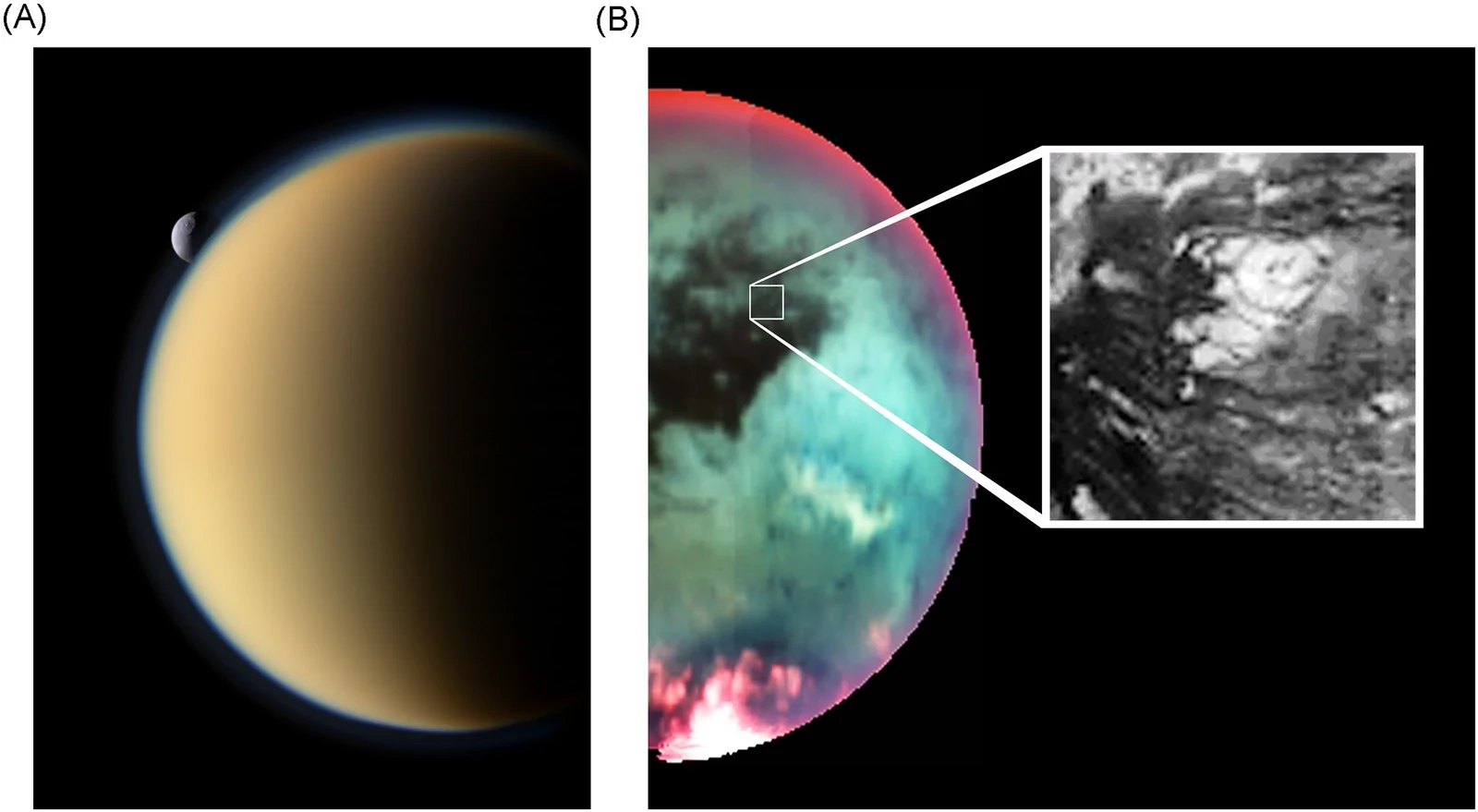
Titan is an abode of NH_3_. (A) Titan captured by the Cassini Imaging Science Subsystem. The thick atmosphere of Titan is visible by a haze across the surface and a lack of surface details. Saturn’s other moon, Tethys, is observed behind Titan. Image credits: NASA/JPL-Caltech/Space Science Institute. (B) False-colour mosaic of Titan obtained by the Visual and Infrared Mapping Spectrometer of Cassini. Colours correspond to atmospheric (red) and surface features (green and blue). The inset image indicates the possible occurrence of a cryovolcano on the surface. Cryovolcanic slurries of water, NH_3_, and methanol have been tentatively observed on the surface. Image credit: NASA/JPL/University of Arizona.

Until Cassini, the surface of Saturn’s largest moon, Titan, was shrouded in uncertainties as its dense atmosphere concealed the surface (Fig. 4A). Earth observations indicated a dense atmosphere consisting of methane (Kuiper 1944). The atmosphere, denser than that of Earth’s, was unlike that observed on any other known moon, and this motivated inspection by the Voyager 1 spacecraft in 1980 (Coustenis 2014). The discoveries made by Voyager 1 revealed a thick nitrogen-methane atmosphere with hydrocarbons and a surface entirely concealed by haze (Smith et al. 1982). But in 2005, close flybys of the surface by Cassini unveiled what was long hidden: a hydrologic-like cycle with methane and ethane, resulting in liquid hydrocarbon clouds, rivers, lakes and seas, and methane rainfalls (Stofan et al. 2007, Turtle et al. 2009, Poggiali et al. 2024), as well as a dynamic surface decorated with drainage networks, fluvial channels (Hörst 2017), mountains (Radebaugh et al. 2007), and possible cryovolcanoes (Lopes et al. 2007, Hörst 2017). The mission also provided evidence of a subsurface ocean below the ice crust (Lorenz et al. 2008, Bills and Nimmo 2011).

A subsurface ocean on Titan had been proposed long before Cassini. The persistence of methane in the atmosphere of Titan, despite continual elimination by photochemical processes, suggested the presence of an internal replenishment mechanism. Lunine and Stevenson (1987) proposed that Titan harboured a subsurface water ocean maintained in the liquid state by the antifreeze properties of NH_3_. In this model, methane retained during Titan’s accretion was thought to be stored in clathrate hydrates (i.e., crystalline structures including water) and periodically released to the surface by cryovolcanic eruptions. Lunine and Stevenson (1987) presented an ammonia–water phase diagram that indicated an ocean of 10%–15% NH_3_ by weight would be required to maintain the liquid state under Titan conditions. Indeed, cosmochemical models indicate that Titan accreted with solar volatiles and may have incorporated significant levels of NH_3_ into its interior (Mousis Olivier et al. 2009). The discovery of NH_3_ on Enceladus by Cassini circumstantially supports the presence of NH_3_ on Titan (Waite et al. 2009). As on Enceladus, further indirect evidence of liquid water in contact with the silicate core in Titan’s history was provided by the detection of ^40^Ar in the atmosphere (Niemann et al. 2005), and an aqueous NH_3_ subsurface ocean on Titan could be a plausible source of atmospheric nitrogen (Owen 2000).

However, the existence of a global liquid reservoir within Titan is currently debated. Recent work suggests Titan’s interior may instead contain high-pressure ice phases and localized, partially melted regions rather than an extensive liquid ocean (Petricca et al. 2025). Such environments are likely characterized by low temperatures and high pressures that may be unfavourable for life as we know it. Consequently, the NH_3_-based habitability considerations discussed in this review apply only to scenarios in which liquid or partially liquid ammonia–water phases are present within Titan’s interior.

In alignment with internal NH_3_ models, cryovolcanic flows observed by the Cassini Titan Radar Mapper exhibited rheological properties consistent with aqueous NH_3_, and possibly methanol, slurries (Fig. 4B) (Lopes et al. 2007, Mitri et al. 2008). However, more recent models place the abundance of NH_3_ at 1.5–5%. A lower NH_3_ concentration fits the observed bulk density of Titan (Tobie et al. 2012, Vance et al. 2018), thermal profiles and mechanical behaviour of the ice shell (Sohl et al. 2014), and the tidal deformation data provided by Cassini (Leitner and Lunine 2019). In addition to NH_3_, radiogenic heating may support the liquid state in Titan’s ocean (Grasset et al. 2000, Sohl et al. 2014). The pH of Titan’s ocean remains undetermined, but is typically modelled at greater than pH 7.3 (Marion et al. 2012, Brassé et al. 2017, Leitner and Lunine 2019). In an ocean of 5% NH_3_, a pH of 11.83 is expected (Brassé et al. 2017). Lower temperatures increase pK_a_ (Zahn 2017a, 2017b, Samuelsen et al. 2019), and Titan’s ocean could be −18°C (Sohl et al. 2014). As NH_3_ dominates above pH 10.1 at 0°C (Bates and Pinching 1949), the ocean of Titan would be expected to bear considerable quantities of NH_3_ compared to NH_4_^+^ under these conditions.

Currently, the presence of a subsurface ammonia–water ocean is largely accepted when modelling Titan’s interior (Grasset and Sotin 1996, Fortes 2000, Grasset et al. 2000, Tobie et al. 2005), and found probable by Cassini’s gravitational data (Goossens et al. 2024). But it is geochemically plausible that NH_3_ dissolved in aqueous solution would react with internal silicates to yield ammonium salts (Kargel 1992, Marion et al. 2012). Indeed, primordial NH_3_ inside Titan could react with sulphate produced by water-rock interactions in the rocky core, forming ammonium sulphate [(NH_4_)_2_SO_4_] rather than remaining as free NH_3_. An aqueous (NH_4_)_2_SO_4_ ocean would have a density capable of generating Rayleigh–Taylor instabilities within the overlying ice crust, potentially driving the rise of buoyant diapirs as a mechanism for cryovolcanism (Fortes et al. 2007, Grindrod et al. 2008). The presence of (NH_4_)_2_SO_4_ would indicate an oceanic pH less than 10.8, which does not align with current estimations. However, salinity and pressure can increase pK_a_ (Clegg and Whitfield 1995, Samuelsen et al. 2019), and thus the pK_a_ of the $N{{H}_3} \rightleftharpoons NH_4^{\ + }$ equilibrium and the pH required for the $NH_4^{\ + }\rightarrow N{{H}_3}$ transition. These effects could shift the equilibrium toward NH_4_^+^ under high pressure and saline conditions, supporting the persistence of NH_4_^+^ despite the high pH of the ocean. Indeed, a pressure of 800 MPa is estimated at the seafloor (Journaux et al. 2020).

Freezing point depression can also be achieved without NH_3_. One of the factors preserving the liquid state of Titan’s ocean could be high salt concentrations (Mitri et al. 2014). The possibility of a magnesium sulphate (MgSO_4_) ocean has been considered, and models of a MgSO_4_ ocean align well with Titan’s density (Vance and Brown 2013, Mitri et al. 2014, Vance et al. 2018). Accretion with MgSO_4_ is plausible as C1 and C2 carbonaceous chondrites contain up to 10% MgSO_4_ (Fredriksson and Kerridge 1988). However, the atmosphere and surface of Titan exhibit reducing conditions. In addition to methane, a suite of reduced organic molecules are present in the atmosphere (e.g., ethane, acetylene, tholins), and there is a lack of oxidants, such as oxygen or carbon dioxide (Kunde et al. 1981, Maguire et al. 1981, Sagan et al. 1992, Niemann et al. 2005, Nixon 2024). Sulphur salts form from oxidation reactions, and thus the redox state of Titan favours NH_3_. Surface analysis by NASA’s upcoming mission to Titan, Dragonfly, may provide clarification into the subsurface ocean composition (Lorenz et al. 2018, Barnes et al. 2021).

## Ammonia in the solar system

Enceladus and Titan represent icy moons of Saturn for which extensive data has been gathered. However, it is not without mention that the Galilean moons of Jupiter’s system, Europa, Ganymede, and Callisto, are also subjects of scientific interest. Europa was first observed from a telescope by Galileo in 1610 (Soderblom 1980). Subsequent magnetic field measurements of Europa by the Galileo spacecraft were consistent with a saline liquid layer (Khurana et al. 1998), and further surface features revealed the presence of mobile icebergs and ice diapirs (Pappalardo et al. 1998, Rathbun et al. 1998, Singer et al. 2021, Kihoulou et al. 2025, Lesage et al. 2025). As for the other moons, Ganymede is the primary target of ESA’s JUICE (Fletcher et al. 2023, Poulet et al. 2024). Along with Titan, Ganymede and Callisto are the largest known icy satellites, and both moons are expected to feature subsurface oceans or liquid reservoirs (Zimmer et al. 2000, Kivelson et al. 2002, Saur et al. 2015, Cochrane et al. 2025).

Thermal models indicate that the Galilean satellites likely contain an NH_3_-rich liquid water layer (Lewis 1971). However, water or salt brines rather than ammoniacal mixtures likely drive cryovolcanism on Europa (Kargel 1991). This is because, unlike the colder Saturn nebula, the thermal properties of the Jovian nebula would have favoured NH_3_ gas. Gas is not well incorporated into smaller bodies during accretion, thus preventing significant incorporation of gaseous NH_3_ into the Jovian moons, including Europa (Kargel 1991, Carlson et al. 2009). Indeed, nitrogen-bearing species have yet to be conclusively detected on Europa, and evidence of tidal heating indicates an antifreeze component such as NH_3_ would not be required to maintain liquid water (Carr et al. 1998, Chen et al. 2014, Běhounková et al. 2021). On the contrary, Ganymede and Callisto formed in the outer, cooler regions of the Jovian disk, where the incorporation of frozen NH_3_ could be plausible (Mousis and Gautier 2004, Mousis and Alibert 2006). Indeed, there is no strong evidence for tidal heating on either of these Jovian moons that could act to preserve liquid water. An antifreeze component within the waters, such as NH_3_, is therefore hypothesized (Khurana et al. 1998, Mousis et al. 2002, Spohn and Schubert 2003, Vance et al. 2018).

Ammoniacal liquid reservoirs are not confined to the icy moons of Jupiter and Saturn, and are hypothesized across a wide range of Solar System bodies. For example, an ocean is expected on Neptune’s moon, Triton. Thermal-structural models of Triton indicate the strong possibility of a long–lived subsurface ocean, probably enriched in NH_3_. However, the presence of NH_3_ in this ocean has not yet been directly confirmed (Gaeman et al. 2012). Similarly, Charon, Pluto’s largest moon, and the moons of Uranus (Ariel, Umbriel, Titania, and Oberon) may also host internal liquid water oceans with NH_3_ (Brown and Calvin 2000, Cheng et al. 2014, Rhoden et al. 2015, Cochrane et al. 2021, Castillo‐Rogez et al. 2023).

As for other planetary bodies, there is evidence of subsurface brines on the Dwarf planet Ceres. Dawn gravity and spectral data indicate localized subsurface brine reservoirs feeding bright surface deposits, and a surface dominated by ammoniated salts and phyllosilicates (Ammannito et al. 2016, Zolotov 2017, Raymond et al. 2020, Singh et al. 2021, Nathues et al. 2022). Pluto may also host a subsurface liquid water ocean, in which liquid water may be maintained by tidal heating or NH_3_ (Robuchon and Nimmo 2011, Nimmo et al. 2016). Indeed, NH_3_ has been detected on the surface (Dalle et al. 2019). More broadly, several Kuiper Belt Objects are predicted to contain subsurface liquid water oceans, in which NH_3_ may be a constituent (Hussmann et al. 2006, Brown 2012). However, few of these bodies have confirmed surface detections of NH_3_, and the composition and extent of their internal liquids remain uncertain.

While many celestial bodies in the Solar System may host liquid water and NH_3_, compositional data is lacking. These limited physicochemical constraints do not allow for the estimation of the abundance, speciation, or phase behaviour of total ammonia, and thus these bodies were not included as part this review.

## An essential ingredient for life

To understand how NH_3_ may influence the habitability of extraterrestrial systems, it is critical to assess how NH_3_ shapes the habitability of terrestrial environments. At 25°C, the $N{{H}_3} \rightleftharpoons NH_4^{\ + }$ equilibrium has a pK_a_ of 9.25, and thus a pH above or below this value (salinity and temperature will also adjust marginally this value) dictates whether NH_3_ (*> \ *pH 9.25) or NH_4_^+^ (*< \ *pH 9.25) predominate (Bates and Pinching 1949, Bower and Bidwell 1978). Many environmental systems are temperate and operate at near-neutral pH and standard atmospheric pressure. Thus, in most water, soil and living systems on Earth, NH_3_ occurs in gas form. However, the natural levels of NH_3_ in water and soil (excluding air) are trace amounts (*< *6 ppm, ≈ 0.00035 M) (Roney et al. 2004). As such, there are limited natural environments that mirror a cold and saline aquatic environment with NH_3_ present, as is speculated in icy moons. There are, however, a few cases where life has documented to thrive in high ammoniacal environments. In bat caves, decomposition of bat urea drives high concentrations of gaseous NH_3_ (McFarlane et al. 1995). Yet microbes, along with bats, colonize this habitat (Studier 1966, Leon et al. 2018, Newman et al. 2018). Similarly, in the alkaline and high pH waters of Mono Lake, United States, concentrations of dissolved NH_3_ gas incrementally increase with depth to a final concentration near 0.0005 M at 35 m. Despite high NH_3_ concentrations, bacteria have been isolated at these depths (Ward et al. 2000, Humayoun et al. 2003). Both examples indicate that life can persist in concentrated NH_3_ environments on Earth.

It is even speculated that NH_3_ may have facilitated the origin of life on Earth. The origin of life is hypothesized to have arisen from increasingly complex interactions that led to the formation of functional cellular bodies (Monnard and Walde 2015, Goldman 2023). It is known that nitrogen is a key element involved in this process as it is required to produce amino acids in modern systems (Miflin and Lea 1982, Bender 2012). In primordial systems, alkaline hydrothermal vents may have acted as locations for prebiotic chemistry as these sites provide heat, chemical gradients, and mineral catalysts (Martin and Russell 2006, Russell et al. 2010, Lane and Martin 2012, Sojo et al. 2016). It is hypothesized that NH_3_ produced in early hydrothermal vents could have been the nitrogen source for prebiotic chemistry (Martin et al. 2008, Sojo et al. 2016, Nishizawa et al. 2021). Indeed, NH_3_ has been demonstrated to form amino acids under early Earth conditions (Miller 1955, Lowe et al. 1963, Furukawa et al. 2009), as well as those present on Titan (Neish et al. 2009, 2010). Very low partial pressures of NH_3_ would have been sufficient to sustain prebiotic chemistry in a seawater origin of life scenario (Wigley and Brimblecombe 1981).

It is of interest to note that hydrothermal-derived NH_3_ could have been prevalent as part of the primordial atmosphere within the Hadean era. The concentration of NH_3_ in the atmosphere dwindled in the Archean era, when the earliest known life existed, as conditions became more oxidizing, and the rate of NH_3_ photodissociation increased as a result of increasing solar luminosity (Brandes et al. 1998, Nishizawa et al. 2021, Shang et al. 2023a, 2023b). It is possible that the heightened levels of NH_3_ in the Hadean era may have been one of the factors that precluded the formation of life earlier in Earth’s history. As a nucleophile, NH_3_ could destroy prebiotic intermediates such as nucleotides and sugars. Indeed, DNA damage can occur with NH_3_ exposure (Zhang et al. 2020, Tong et al. 2025). As a H^+^ acceptor, NH_3_ could also act to limit the H^+^ pool for prebiotic chemistry (Aithal et al. 2023, Pathak et al. 2024). Consequently, elevated NH_3_ in the Hadean era may have functioned as a chemical barrier to early prebiotic pathways, with its eventual reduction opening the window for life’s emergence in the Archean era.

In modern systems, total ammonia sustains habitability by forming a vital part of the nitrogen fixation cycle. NH_3_ is formed naturally by diazotrophic bacteria, cyanobacteria, and archaea that perform nitrogen fixation. In this process, atmospheric nitrogen (N_2_) is reduced to NH_3_ (Burris and Roberts 1993, Ribbe 2011, Shin et al. 2016). NH_3_ may also be available as a result of ammonification by decomposition of organic excretion or tissue (including DNA, proteins, and amino acids) by various fungi and prokaryotes (Ladd and Jackson 1982, Strock 2008, Singh K 2016). However, most of the NH_3_ produced rapidly equilibrates to NH_4_^+^ due to the moderate pH of many ecosystems (Fowler et al. 2013). At this stage, the total ammonia in an environment may be directly assimilated into microbes and plants and incorporated into the synthesis of amino acids and nucleotides, whereby glutamate, a nitrogen donor, is the first and most central amino acid formed from NH_4_^+^ (Rogers and Aneja 1980, Smith et al. 1980, Tesch et al. 1999, Vo et al. 2013, Hachiya and Sakakibara 2017). Alternatively, the pool of NH_4_^+^ and NH_3_ in the environment is oxidized by nitrifying micro-organisms such as ammonia-oxidizing bacteria (AOB) to nitrite, which is subsequently oxidized to nitrate by nitrite-oxidizing bacteria (Wallace and Nicholas 1969, Schmidt and Belser 1983, Koops and Pommerening-Röser 2001, Caranto and Lancaster 2017). The nitrogen cycle is completed by denitrifying bacteria such as *Paracoccus* and *Pseudomonas* that process nitrate back into N_2_ (Alexander 1965, Ji et al. 2015, Rajta et al. 2020). The presence of NH_3_ and NH_4_^+^ in the environment thus plays a crucial role in supporting diverse microbial communities and plants by ensuring the continuous availability of biologically usable nitrogen.

## Biochemical disruption and disorder

While total ammonia forms a vital part of the global nitrogen cycle, high concentrations of NH_3_ are widely documented as toxic to life as we know it. The small size and uncharged nature of gaseous NH_3_ permits passive, unregulated permeation across lipid membranes (Ritchie and Gibson 1987a, 1987b, Ritchie and Islam 2001, Brazier 2016). Due to the lone pair of electrons on the nitrogen atom, NH_3_ may function as a H^+^ acceptor. Under biological pH (i.e., pH 7.4 to 7.8), permeated NH_3_ combines with cytoplasmic H^+^ to form NH_4_^+^. The capture of H^+^ increases cytosolic pH (Bosoi and Rose 2009).

**Figure 5:**
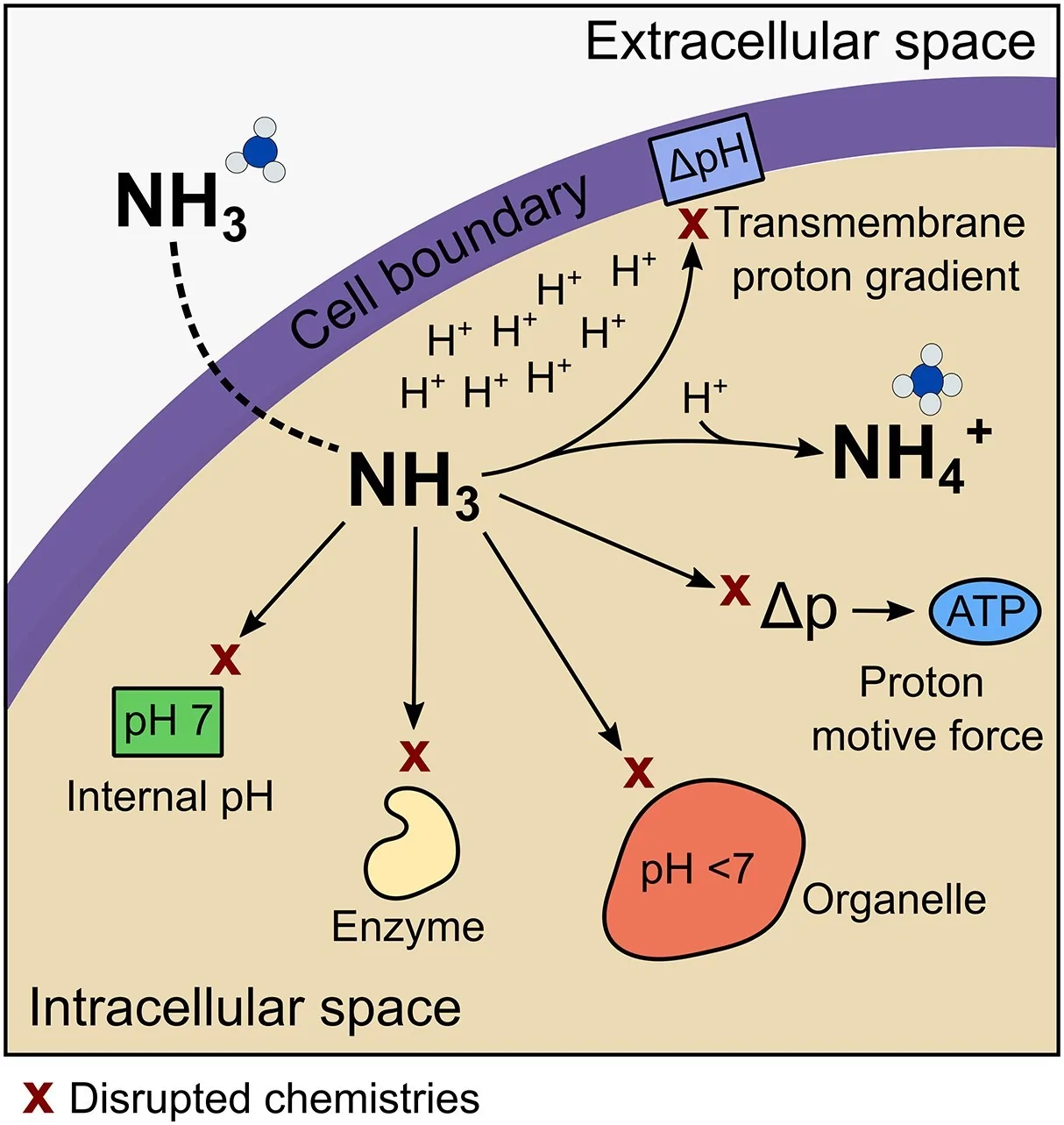
Mechanisms of cellular NH_3_ toxicity. Research across the domains of life has revealed several pathways of cellular NH_3_ toxicity caused by disrupted internal chemistries. These toxic effects are exerted firstly by the disruption of the intracellular proton pool, as the conversion of $N{{H}_3}\rightarrow NH_4^{\ + }$ occurs following unregulated, passive diffusion of NH_3_. Downstream chemistries such as intracellular pH, the transmembrane proton gradient, and proton motive force can become disrupted as a result. Enzyme and organelle function may also become disrupted by changes in pH.

In eukaryotic cells, such intracellular NH_3_-driven alkalization has been associated with disrupted calcium (Ca^2+^) signalling (Horie et al. 1995, Rose et al. 2005), disrupted organelle acidification, and with enzyme dysfunction (Moriwaki et al. 2024). Additionally, capture of H^+^ can dissipate the proton motive force required for ATP generation (Bai et al. 2001, Angelova et al. 2022), causing oxidative stress by the production of reactive oxygen species (Han et al. 2020, Angelova et al. 2022). In prokaryotes, specific effects of NH_3_-driven reactions remain poorly characterized, but NH_3_ transport has been correlated with the dissipation of the transmembrane pH gradient in *Methanospirillum hungatei* (Sprott et al. 1984). Increased NH_4_^+^ and NH_3_ have also been correlated with reduced ATP production and electron transport system activity in *Enterobacter cloacae* HNR (Weng et al. 2022). In general, internal alkalization can elicit a stress response in prokaryotes (Schuldiner et al. 1986). These cellular toxicity mechanisms are depicted in Fig. 5. Both higher and lower organisms typically prevent NH_3_ toxicity and accumulation by rapid assimilation, enhanced excretion, conversion to less toxic compounds, or scavenging (Givan 1979, Burkovski 2003, Ip and Chew 2010, Chew and Ip 2014, Haskett et al. 2022, Okabe et al. 2023, Kang et al. 2026). For example, upon detecting NH_3_, *Burkholderia glumae* triggers oxalate biosynthesis that can act to neutralize NH_3_ (Kang et al. 2026). However, in high enough concentrations, NH_3_ may overwhelm these defensive mechanisms.

In addition to internal alkalization, the external rise in pH caused by NH_3_ has also been implicated as the cause of toxicity in living organisms (Vines and Wedding 1960, Taglicht et al. 1987). Indistinguishable toxicity between NH_3_/NH_4_^+^ solutions and solutions of identical pH has been characterized for *Bacillus subtilis, Sporosarcina, Paenibacillus, Staphylococcus, Brevibacillus, Streptomyces, Pseudomonas*, and *Arthrobacter* (Deal et al. 1975, Kelly et al. 2012). In *Escherichia coli*, results vary. In a study by Deal et al. (1975), NH_3_/NH_4_^+^ solutions were shown to be more toxic than NaCl solutions at an equivalent pH of 9.5, but indistinct toxicity was observed at pH 10 between the NH_3_/NH_4_^+^ solutions and NH_3_-free solutions at comparable pH. Likewise, for *Bacillus subtilis*, toxicity between NH_3_/NH_4_^+^ solutions and NaCl solutions was distinct at pH 9 but indistinct at pH 10 (Deal et al. 1975). Vines and Wedding (1960) indicated that, in plants, high pH is not a direct toxicity mechanism of NH_3_ per se, but rather a vehicle through which larger amounts of gaseous NH_3_ can enter cells and disrupt biological processes. Similarly, in the alkalitolerant extremophile *Halomonas meridiana*, growth in NH_3_/NH_4_^+^ solutions with a high relative abundance of NH_3_ and pH-matched solutions of sodium hydroxide indicated that NH_3_ toxicity is distinct from external pH stress (Hopton et al. 2025a). This study also elucidated that NH_3_ induced alterations to metabolites that could suggest cell wall modification, alterations to metabolites within Coenzyme A pathways, and accumulation of compounds that could reflect internal NH_3_-driven reactions (Hopton et al. (Hopton et al. 2025a).

In contrast to NH_3_, the positive charge of NH_4_^+^ prevents passive diffusion through the hydrophobic core of lipid membranes. Instead, NH_4_^+^ is transported through ammonium transport (Amt) membrane proteins (Kim et al. 2012, Wacker et al. 2014), which may also facilitate passive NH_3_ diffusion (Soupene et al. 1998, Soupene et al. 2002). Due to similarities in charge, ionic radius, and hydration properties, NH_4_^+^ can compete with potassium ions (K^+^) for transport through ion channels (Moser 1987, Neijssel et al. 1990, Bosoi and Rose 2009). This facilitated diffusion and active transport of NH_4_^+^ regulates cellular uptake, thereby reducing toxicity of NH_4_^+^ relative to NH_3_. However, high accumulations of NH_4_^+^, such as that caused by cation channel influx (Burckhardt and Frömter 1992, Ramirez et al. 1999) or unregulated permeation of NH_3_, can upset cell homeostasis. NH_4_^+^ can acidify the intracellular medium (Burckhardt and Frömter 1992, Ramirez et al. 1999) and influence membrane potential (Golby et al. 1990, Wang et al. 2018). Elevated NH_4_^+^ can also disrupt K^+^ balance (Sprott and Patel 1986, Szczerba et al. 2008, Kong et al. 2014, Shi et al. 2020), which is essential for pH balance, membrane potential, intracellular communication, electrical signalling, as well as osmoprotection [for extended reviews, see Beagle and Lockless (2021) and Benarroch and Asally (2020) for bacteria, Johnson et al. (2022) for plants, and McLean and Wang (2021) for humans].

The downstream effects of NH_4_^+^ are much more defined in literature across a breadth of species compared to NH_3_. Such effects include disruption of amino acid, lipid, and nucleotide metabolisms in shrimp and bacteria (Xiao et al. 2019), production of reactive oxygen species, interruption of Ca^2+^ homeostasis, and induction of cell apoptosis in mammalian cells (Wang et al. 2018), acidification in plants (Hachiya et al. 2021), and decline of motility and energy metabolism in bacteria (Weng et al. 2022). Bacteria have also exhibited alterations to amino acid levels (Weng et al. 2022, Hopton et al. 2025b) and protein factors involved in translation, translocation, and folding (Sayavedra-Soto et al. 2015, Zorz et al. 2018) under NH_4_^+^ exposure. Accumulating concentrations of NH_4_^+^, as opposed to increasing NH_3_ or pH, have been found to be a contributing factor suppressing the abundance of *Anaerobranca, Tepidimicrobium* and *Proteiniborus* in anaerobic digesters (Dai et al. 2016).

Toxicity occurring by the independent action of NH_4_^+^ is thus possible, although less common due to the regulated transport of this ion. Evidence thus far indicates the overall picture of NH_3_ toxicity is one where both NH_3_ and NH_4_^+^ act in concert against biological components: NH_3_ bypasses membrane regulation and passively diffuses into cells, NH_4_^+^ is formed following the permeation of NH_3_ into cells, and damaging cellular effects occur by the actions of both NH_3_ and NH_4_^+^.

## Biotic models for assessing habitability

Icy moons are, relative to conditions on Earth, extreme environments. There are extremes of temperature and pressure exceeding those even possible on Earth. This includes near −200°C icy surfaces (Brown et al. 2006, Ashkenazy 2019, Jennings et al. 2019) and oceanic pressures of 800 MPa (Journaux et al. 2020). Subsurface waters could fall between a salinity of 2 and 20 g/kg on Enceladus, but up to 200 g/kg on Titan. Comparatively, the salinity of seawater on Earth is ∼35 g/kg or 3.5% (Ludwig 2022). The waters of Enceladus and Titan are also expected to exceed a pH of 10 (Brassé et al. 2017, Glein and Truong 2025). Organisms that could develop under these extremes of pH, temperature, salinity, and pressure would have to utilize specialized adaptations to survive. On Earth, such organisms are known as extremophiles. Extremophiles on Earth already demonstrate that life can survive in waters at −20°C (Clarke et al. 2013, Frösler et al. 2017), pH 11 (Suzuki et al. 2014), and upwards of 200 g/kg salinity (Meinzer et al. 2023), for example.

Given the ammoniacal waters of icy moons, it is notable that some prokaryotes on Earth display NH_3_-dependent metabolisms. These organisms include ammonia-oxidizing archaea (AOA) and AOB, chemolithotrophs that utilize NH_3_ as an energy resource by oxidizing NH_3_ to nitrite in the first-rate limiting step of nitrification (Koops et al. 2006, Zorz et al. 2018). However, *-phile* often implies thriving in highly concentrated or extreme conditions. AOA and AOB are tolerant to high NH_4_^+^ (0.01 M to 0.43 M) but not NH_3_ (activity reduction of 15.9% was observed in less than 0.001 M NH_3_) (Tourna et al. 2011, Vejmelkova et al. 2012, Qian et al. 2017).

When considering the habitability of icy moons, the NH_3_ concentrations estimated for their subsurface oceans are low relative to the inferred bulk chemical compositions. This raises questions as to whether life would need NH_3_ adaptations to evolve and thrive. Indeed, methanogenic archaea, which are typically without adaptations to high NH_3_, are frequently considered one type of metabolic analogue for life within icy moon oceans. Methanogens have been shown to continue weak metabolic functions in NH_3_ levels higher than those expected on Enceladus (≈ 0.0265 M) (Wang et al. 2015). Methanogens have also been isolated from an array of extreme environments on Earth, use simple energy sources (e.g., hydrogen and carbon dioxide) to produce methane (methane has been detected on both Enceladus and Titan), and are anaerobic (McKay et al. 2008, Taubner et al. 2015). Due to limited oxygen delivery to the oceans, anaerobic respiration was thought to be a requirement in the oceans of icy moons. These properties have made methanogens suitable study specimens in astrobiology.

Models now suggest that oxygen generated on the surface of icy moons by radiation is transported to the subsurface oceans (Teolis et al. 2017, Ray et al. 2021, Hesse et al. 2022, Szalay et al. 2024, Tinner et al. 2024). This process has been proposed to broaden the metabolic possibilities in these oceans to aerobes. Thus, given the low bulk compositions of NH_3_ in icy moons, this finding expands biotic models beyond AOB, AOA, or methanogens to also include aerobic extremophilic bacteria that do not have specified NH_3_ adaptations. Extremophilic bacteria capable of both aerobic and anaerobic metabolisms are widely documented in the literature [see Pikuta et al. (2007) or Bowers et al. (2009) for in-depth reviews]. When choosing suitable biotic models for assessing habitability, it must be considered that, unlike methanogenic archaea, bacteria exhibit a broader range of metabolic pathways that may be compatible with icy moon chemistries (e.g., sulphur oxidation, nitrate reduction, NH_3_ oxidation) (Koops et al. 2006, Yin et al. 2014, Kilic et al. 2017). Bacteria lacking specific NH_3_ adaptations have also been isolated at depths of 35 m in the NH_3_-laden (0.0005 M NH_3_) Mono Lake, as aforementioned (Humayoun et al. 2003), as well as the hypersaline and −13°C waters of the ice-sealed Lake Vida (Murray et al. 2012). The use of extremophilic, and possibly aerobic, bacteria without specified NH_3_ adaptations in limits-of-life research could therefore offer a nuanced perspective on the habitability potential of icy moons.

Using bacteria as model organisms, the following sections examine whether the ammoniacal subsurface oceans of icy moons could sustain life by drawing on known bacterial survival thresholds in NH_3_.

## Bacteria under simulated icy moon extremes

As life could be entrained in the ejected ice grains from the plumes of Enceladus, much microbiology research in astrobiology has focused on viability and detectability of microbes following simulated entombment in ice (Kelly et al. 2012, Bywaters et al. 2020, Parker et al. 2023, Klenner et al. 2024). Few experiments have provided *in vitro* assessments of how simulated icy moon conditions with NH_3_ could afflict bacterial life. Those that have, have done so from a planetary protection perspective, assessing the risk of surface contamination on icy moons with common terrestrial bacteria carried by spacecraft, not extremophiles. However, this data can still provide valuable constraints on habitability in NH_3_. Molton and Ponnamperuma (1972) demonstrated survival thresholds of four bacteria, *E. coli, Serratia marcescens, Aerobacter aerogenes*, and *B. subtilis*, to a simulated Jovian atmosphere of H_2_ (56%), He (43%), CH_4_ (0.5%), and NH_3_ (0.5%). Pressure-temperature regimes included those that could apply to icy moon interiors (≈ 5 MPa and −13°C, and ≈ 6.8 MPa and 0°C). Survival varied with these conditions. Near-total mortality occurred under the colder treatment (loss of viability as %: *E. coli*, 97%; *S. marcescens*, 93%; *A. aerogenes*, 63%; *B. subtilis*, 100%), while fewer proportional deaths occurred at 0°C (loss of viability as %: *E. coli*, 19%; *S. marcescens*, 50%). Although not designed to assess oceanic habitability, these findings indicate that microbial persistence in NH_3_ mixtures may be constrained at sub-freezing temperatures or supported at near 0°C.

The multi-extreme conditions employed by Molton and Ponnamperuma (1972) make it difficult to attribute a single parameter, or a combination of parameters, to bacterial death. Specifically, the influence of NH_3_ on bacterial survival limits cannot be established. Subsequent studies, however, have isolated the impact of NH_3_ by examining bacterial viability in simple, aqueous NH_3_/NH_4_^+^ solutions under extreme temperatures. Deal et al. (1975) showed that a 0.1 M NH_3_/NH_4_^+^ solution was toxic to *E. coli* and *B. subtilis* at 25°C and pH 9.5–10.5 (NH_3_  *> * 50%). Molar limits of toxicity were not assessed, but cell viability was reduced at a higher rate as pH increased at 25°C. This correlates to an increased relative proportion of NH_3_. Reduction to cell viability still occurred at 0°C, albeit at a slower rate. From this, we can make two assumptions: abundance of NH_3_ is proportional to toxicity, and lower temperatures reduce toxicity. The latter phenomenon could be explained by a reduction in kinetic energy associated with lower temperatures that ultimately limits substantial membrane permeation of NH_3_. The parameters utilized in this study (i.e., pH 10.5, temperatures at 0°C and 0.1 M NH_3_/NH_4_^+^) align well with the physicochemical properties expected on Enceladus. These findings imply that terrestrial organisms like *E. coli* and *B. subtilis* would face significant physiological stress, if not outright mortality, under such conditions.

While simulated icy moon environments *in vitro* have indicated that the presence of NH_3_ can contribute to the toxicity of an aqueous solution, these experiments do not define the concentration thresholds for life in aqueous NH_3_. As presented in Table 1, estimated concentrations of NH_3_/NH_4_^+^ between icy moons vary. In accordance with the toxicological data, aqueous environments with NH_3_, such as those hypothesized for Enceladus and/or Titan, may present a more significant physiological challenge to microbial survival and adaptation if present at sufficient concentrations. However, the definition of “sufficient concentrations” is loose; the concentration thresholds for the growth of bacteria in NH_3_ have not yet been properly established.

**Table 1:** NH_3_ and NH_4_^+^ concentration and speciation estimated for icy moon subsurface oceans.

Icy moon	Predominant species^1^	Estimated [NH_3_]	Estimated [NH_4_^+^]
Enceladus	NH_3_	0.0181 M^2^	0.00744 M^2^
Titan	NH_3_	0.96 to 9.6 M^3^	Not estimated

1 Speculated in this review as per physicochemical expectations of oceans.

2 Estimated by Glein and Truong (2025).

3 Molarity (M) calculated using an oceanic density (*ρ *) of 1,091 g/l (Goossens et al. 2024), 1.5% to 15% NH_3_, and the molar mass of NH_3_ at 17.031 g/mol (Equation 1).


(1) \begin{eqnarray*}
M = \ \frac{{\rho \ \times \ \% N{{H}_3}}}{{\textit{Molar}\ \textit{mass}}}
\end{eqnarray*}

## Ocean habitability: limits of bacterial life in ammonia

There is not a significant body of work that intersects microbiology, icy moon physicochemical conditions, and NH_3_. In lieu of this, survival thresholds in NH_3_/NH_4_^+^ can be gleaned from research in other fields. For example, much existing work regarding survival thresholds of bacteria in NH_3_ has been conducted from the perspective of wastewater treatment in anaerobic digesters. Anaerobic digesters are often maintained below pH 9.25, whereby the relative abundance of NH_3_ is less than 50% compared to NH_4_^+^ (Dai et al. 2016, Jiang et al. 2019, Mutegoa et al. 2020, Chapleur et al. 2021). These conditions are not comparable with those estimated in the oceans of Enceladus and Titan. However, concentration limits of bacterial life in NH_3_ can still be drawn from this research.

Considering this, the minimal inhibitory concentration (MIC) for a wide variety of bacteria in NH_3_ and NH_4_^+^ is presented in Table 2. Where MIC was not stated outright, an estimate was derived from the pH of the experiment using the pK_a_ of $N{{H}_3} \rightleftharpoons NH_4^{\ + }$ and the Henderson–Hasselbalch equation. In these instances, the MIC was defined as the lowest concentration of NH_3_ or NH_4_^+^ that limited visible growth and function.

**Table 2:** Minimal inhibitory concentration (MIC) of NH_3_ and NH_4_^+^ across bacterial species.

Species	Strain	MIC (NH_3_, M)	MIC (NH_4_^+^, M)
*Enterobacter cloacae* ^a^	HNR	≈ 0.00 049	≈ 0.0138
*Escherichia coli* ^b^	MG1655	0.0042	0.750^§^
*Corynebacterium glutamicum* ^b^	ATCC 13 032	0.0112	1.989^§^
*Bacillus subtilis* ^b^	–	0.0133	0.750^§^
*Escherichia coli* ^c^	K-12	*> \ * 0.02, *< * 0.04^†^	–
*Bacillus subtilis* ^c^	168	*> \ * 0.02, *< * 0.04^†^	–
*Enterococcus durans* ^c^	ST2	*> \ * 0.02, *< * 0.04^†^	–
*Pseudomonas* sp.^c^	–	*> \ * 0.02, *< * 0.04^†^	–
*Enterobacter faecalis* ^d^	NCTC 00 775	0.025	0.465
*Listeria innocua* ^d^	NCTC 11 288	0.025	0.465
*Escherichia coli* ^d^	NCTC 10 538	0.025	0.465
*Halomonas meridiana* ^e^	Sltfh1	0.03	0.02
*Bacillus subtilis* ^d^	T1, T2, T19, T22, T34, T39, DK-W1, N3, N4 and N5	0.05	0.931
*Bacillus cereus* ^d^	T31 and T38	0.05	0.931
*Bacillus megaterium* ^d^	T3, T4, T21, T34, T37 and T40	0.05	0.931
*Pseudomonas aeruginosa* ^d^	NCTC 10 299	0.05	0.931
*Bacillus subtilis* ^f^	168	≈ 0.064	≈ 1.456
*Bacillus subtilis* ^d^	T5 and DA2	0.15	0.279
*Bacillus cereus* ^d^	NCIMB 9373	0.15	0.279
Sulphate-reducing bacteria^g^	–	0.2^‡^	0.166^‡^
Bacterial isolate^c^	4–1	≈ 0.3^†^	–
Bacterial isolate^c^	4–2	≈ 0.3^†^	–
*Bacillus subtilis* ^d^	T20, N1, N2, DA1 and NCIMB 3610	0.3	0.559
*Bacillus cereus* ^d^	T41	0.3	0.559
*Bacillus licheniformis* ^d^	ATCC 39 302	0.3	0.559
*Enterococcus faecium* ^d^	DK-C1	0.3	0.559
*Micrococcus luteus* ^d^	NCDO 0982	0.3	0.559
*Staphylococcus aureus* ^d^	NCDO 0949	0.3	0.559
*Salmonella typhimurium* ^d^	NCIMB 10 248	0.3	0.559
*Bacillus subtilis* ^d^	T36	0.5	0.931
*Proteus morgani* ^d^	NCIMB 00 067	0.5	0.931
*Bacillus pumilus* ^d^	NCIMB 9369	*> \ * 0.5	0.931
*Bacillus pasteurii* ^d^	NCIMB 8841	*> \ * 0.5	0.931

a From Weng et al. (2022).

b From Müller et al. (2006).

c From Tada et al. (2021).

d From Leejeerajumnean et al. (2000).

e From Hopton et al. (2025a).

f From Hamill et al. (2020).

g From Dai et al. (2017).

§ Growth impairment attributed to osmotic or ionic effects of NH_4_^+^.

† Cultured in NH_3_ gas.

‡ MIC derived from NH_3_ and NH_4_^+^ concentrations in reactor 3.

**Figure 6:**
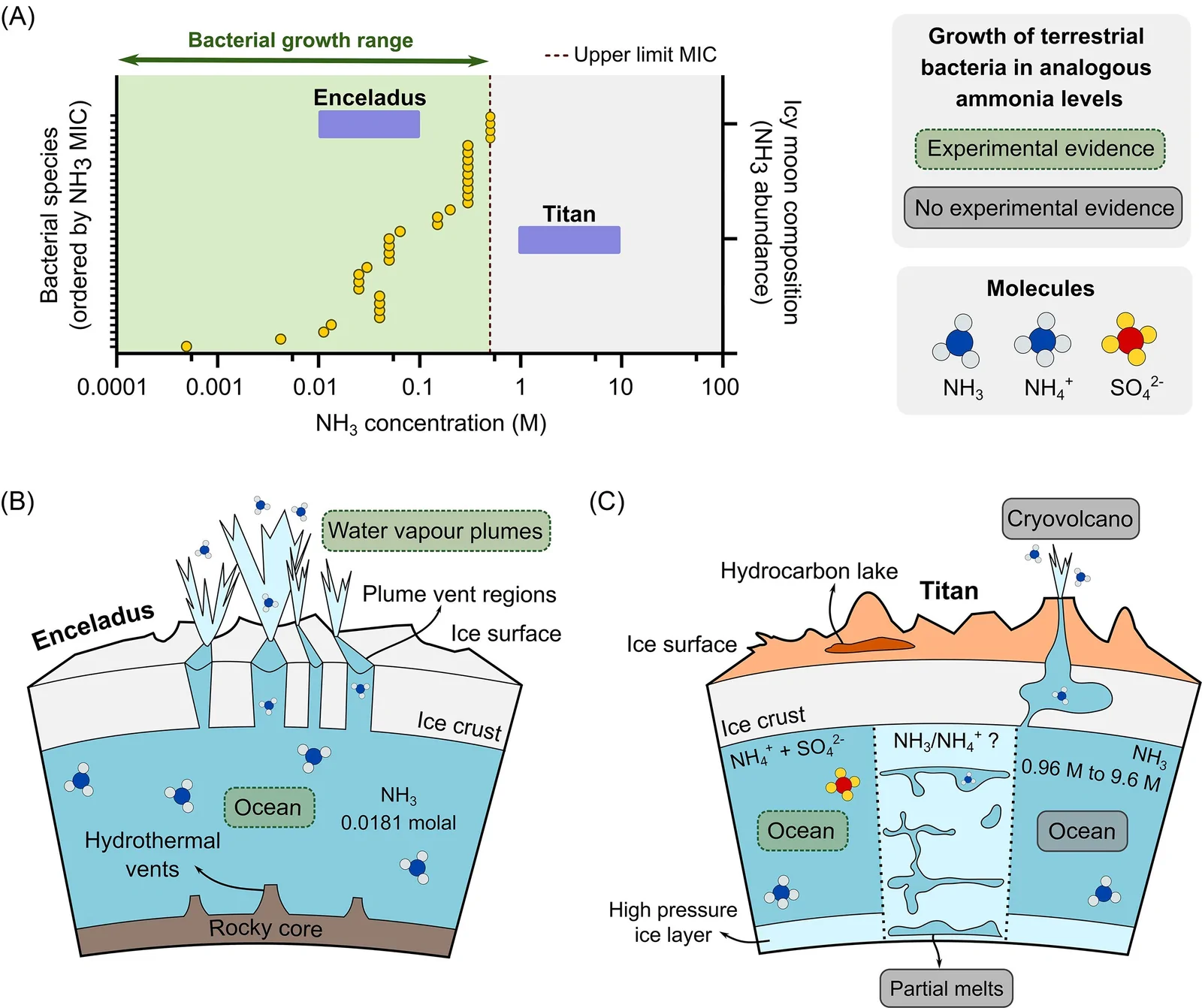
Survival of terrestrial life in NH_3_ paralleled against known ammoniacal environments on icy moons. (A) Minimal inhibitory concentrations (MIC) of NH_3_ in bacteria from Table 2 compared against estimated NH_3_ concentrations within the oceans of Enceladus and Titan. NH_3_ concentration on the *x*-axis represents the MIC for bacteria, and the ocean abundance for icy moons. The red dotted line indicates the upper limit of observed bacterial MIC in NH_3_. (B, C) Environments where growth of terrestrial bacteria in similar NH_3_ or (NH_4_)_2_SO_4_ levels have been experimentally demonstrated (green, dashed line) or not yet demonstrated (grey, solid line) *in vitro* are depicted. (B) Schematic overview of the exterior and interior of Enceladus. Subsurface ocean NH_3_ concentration determined by Glein and Truong (2025). Speciation speculated as part of this chapter based upon estimated physicochemical characteristics of the ocean. (C) Cross section of Titan. Left: A putative global subsurface ocean enriched in ammonia–water brines, potentially containing ammonium salts such as (NH_4_)_2_SO_4_, within which terrestrial bacteria have demonstrated high tolerance. Centre: an alternative interior model in which Titan consists of a slushy high-pressure ice layer with small amounts of partial melting. NH_3_ and NH_4_^+^ concentrations are currently unconstrained. Right: a Titan model with a global subsurface ocean that could be comprised of 1.5%–15% NH_3_, calculated at a molarity of 0.96–9.6 M as part of this review.

From Table 2, it is evident that the NH_3_ concentrations estimated on Enceladus fall below the MIC of NH_3_ established for many of the bacteria. The intersection between established bacterial MIC of NH_3_ and icy moon NH_3_ levels is depicted in Fig. 6A. The exceptions are *E. cloacae* HNR, *E. coli* MG1655, *B. subtilis*, and *Corynebacterium glutamicum* ATCC 13032, which exhibit sensitivity to concentrations of NH_3_ below 0.0181 M. It is notable the maximal inhibitory threshold for *B. subtilis* is 0.5 M. Spore-formation of *B. subtilis* has been implicated for the survival of this species when frozen in 35% NH_3_ (Kelly et al. 2012). *B. pumilus* and *B. pasteurii* have demonstrated a tolerance up to, but not necessarily limited to, 0.5 M NH_3_, the current upper limit of bacterial survival in NH_3_ recorded in literature (Leejeerajumnean et al. 2000). The high level of tolerance could be attributed to NH_3_ utilization; in *B. pasteurii*, NH_3_ supports substrate oxidation (Wiley and Stokes 1962), permeability of substrates (Wiley and Stokes 1963), and ATP generation (Jahns 1996). These comparisons indicate that the levels of NH_3_ speculated in the ocean and plumes may not limit prospects for habitability (Fig. 6B). However, it should be noted that the survival limit of 0.5 M NH_3_ has only been identified in one study. This study did not monitor NH_3_ levels, and the result has not been substantiated by any further studies.

Nonetheless, the survival of *B. pumilus* and *B. pasteurii* in up to 0.5 M NH_3_, the maximum concentration utilized in the study by Leejeerajumnean et al. (2000), suggests survival could possibly exceed 0.5 M NH_3_. This could indicate concentrations of 0.96 M NH_3_ (Table 1), tentatively calculated within the putative ocean of Titan, could be survived by some species. However, survival in 0.96 M NH_3_, or concentrations exceeding 0.5 M NH_3_, has not been explicitly demonstrated. Thus, based on the data presented in Table 2, the known NH_3_ tolerance in bacteria is lower than the NH_3_ concentrations estimated on Titan (Fig. 6A). This limits habitability prospects in the ocean or likely any adjoining cryovolcanoes (Fig. 6C). However, if it is true that (NH_4_)_2_SO_4_ predominates in the ocean, the levels of NH_4_^+^ could fall within a range that has been observed to support bacterial survival of Earth. Survival has been documented in up to 1.989 M NH_4_^+^ in *C. glutamicum*. The concentration of (NH_4_)_2_SO_4_ under the aqueous (NH_4_)_2_SO_4_ ocean model has not been estimated. However, if we assume NH_3_ incorporation during accretion was similar under the two models, concentration of (NH_4_)_2_SO_4_ may be comparable. As such, lower threshold levels of 0.96 M to 2 M (NH_4_)_2_SO_4_ may not pose a barrier to habitability.

We paint a broad picture of habitability here. However, habitability in ammoniacal environments may be enhanced locally through habitat modification. For example, microenvironments could develop where microbial activity lowers pH, shifting the NH_3_/NH_4_^+^ equilibrium toward NH_4_^+^, which is less toxic. Additionally, microbes may utilize available NH_3_ for biosynthesis, exporting amino acids, or other metabolic processes. These scenarios suggest that native life on such worlds could possess physiological adaptations conferring NH_3_ tolerance, highlighting that local chemical modifications and evolutionary adaptations may create habitable niches even in waters that are overall NH_3_-rich.

It should be noted that while the physicochemical conditions underpinning the MIC of NH_3_ and NH_4_^+^ given in Table 2 may not be entirely comparable to icy moons, some of the bacteria utilized exhibit polyextremophilic attributes (*H. meridiana*), alkaliphilic and halotolerant metabolisms (*B. licheniformis*), or alkalitolerance and halotolerance (*B. subtilis, B. cereus, E. faecium, E. durans, M. luteus*) that are relevant to the high pH and saline properties expected in icy moon oceans. It should be additionally stated that the MIC of NH_3_ is not an absolute limit to life. Bacteria could persist in a dormant, non-growing but viable state. However, for the purpose of this review, we consider habitability on the basis of whether an environment can: a) support the emergence of life, and b) support replication and growth. As such, the preceding comparison was not meant to imply the presence of life but rather explore whether the likelihood of habitability could be inferred by comparing the known biological limits in NH_3_ with extraterrestrial ammoniacal conditions.

## Habitability of ammoniacal ice

Oceans are not the only environments within icy moons that could hold water, and thus, potential habitats. The ice shells of icy moons could support brine channels, veins, pockets, and fractures (Kargel et al. 2000, Buffo et al. 2021, Wolfenbarger et al. 2022a), Buffo et al. 2023). On Earth, such brine networks have shown to sustain life (Cooper et al. 2019, Buffo et al. 2022). Organisms can be entrained into liquid inclusions of ice shells during ice formation. The subsequent labyrinth of brine networks connected to the ocean below can provide liquid water, nutrients, and dissolved gas that preserve viable life (Dieckmann 2002, Loose et al. 2011). Viruses, prokaryotes, and eukaryotes have been isolated from terrestrial sea ice (Maranger et al. 1994, Brown and Bowman 2001, Lizotte 2003, Mueller et al. 2005). *Chroococcidiopsis* CCMEE 029 and CCMEE 171 have been shown capable of surviving in sodium sulphate (Na_2_SO_4_), Mg_2_SO_4_, and NaCl ice frozen to −40°C (Cosciotti et al. 2019). It has therefore been suggested that cryobrine networks in ice shells of icy moons could offer viable habitats, particularly those near the ice-ocean interface where temperatures are more suitable for biological propagation (Kargel et al. 2000, Wettlaufer 2009). While a majority (∼80%) of these brine habitats on Europa may not supply enough nutrients to become inhabited, it is estimated ∼20% could sustain life without active growth under nutrient-limited conditions. A smaller portion of nutrient-rich brine channels, isolated near the ice-ocean interface, may additionally support active growth (Wolfenbarger et al. 2022a). Such habitable ice environments proximal to the ice-ocean interface have also been suggested on Enceladus (Buffo et al. 2021).

However, brine distribution within icy shells is also influenced by freeze-concentration processes resulting from sills and diapirs (Neveu et al. 2015, Wolfenbarger et al. 2022b). As pure water freezes from ascending ocean-derived melts or intrusions, residual liquids could become increasingly saline and NH_3_-rich, promoting accumulation of dense brines in veins and pockets within the surrounding ice (Neveu et al. 2017). Continued freezing could cause expansion and overpressure, contributing to fracture propagation, cryovolcanic transport, and plume or cryolava emplacement at the surface (Neveu et al. 2015). Such freeze-concentration may, in many settings, drive brine compositions toward high ionic strength and low water activity conditions that are less favourable for life. Consequently, the habitability of ice-shell brine networks likely varies spatially with thermal and emplacement history, and some veins produced by sill and diapir freezing may be comparatively inhospitable despite containing liquid and nutrients.

**Figure 7:**
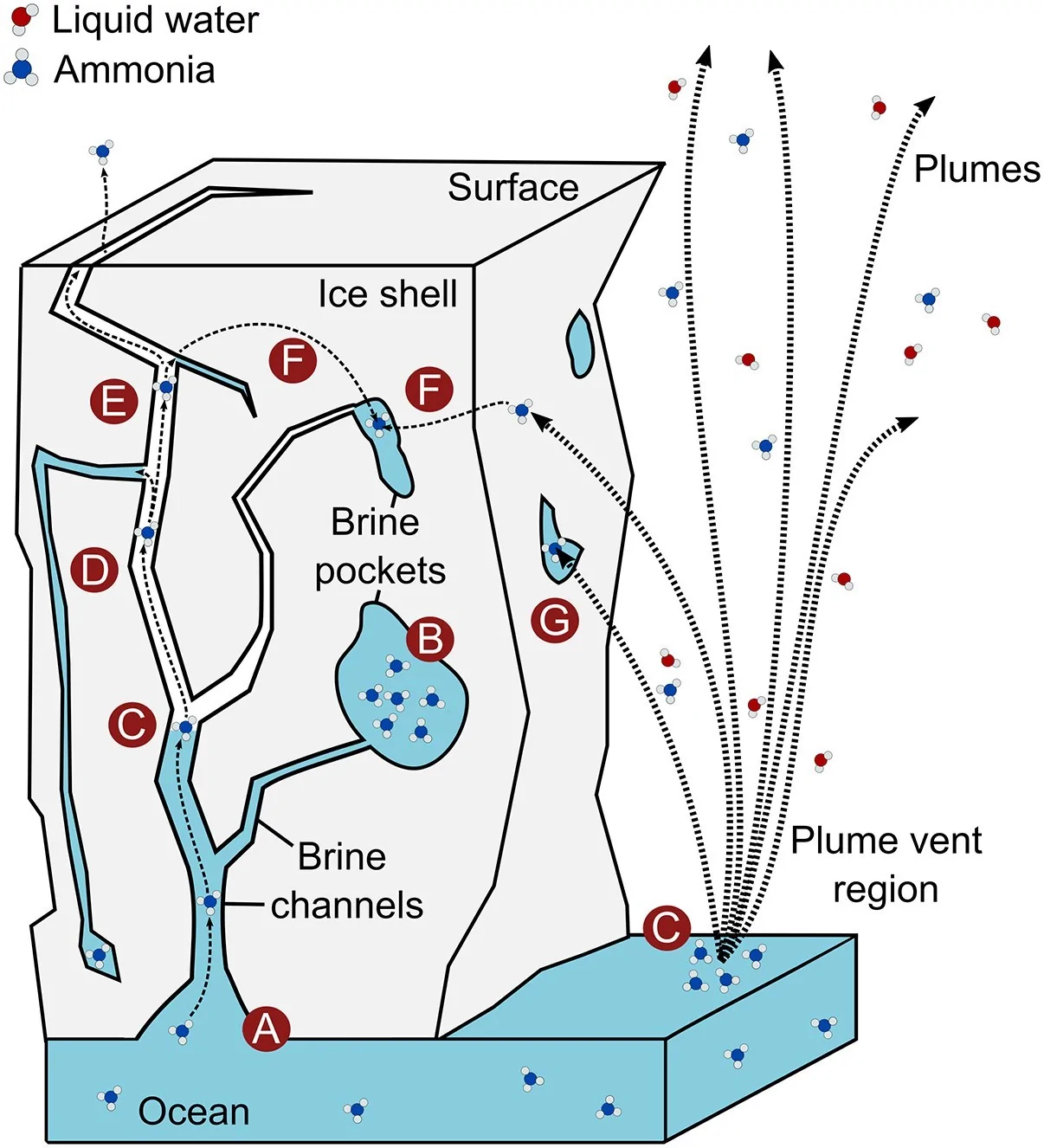
Penetration of NH_3_ into icy moon ice shell habitats. (A) Brine may percolate into channels from the ocean below, delivering chemical constituents, including NH_3_ and liquid water, to these habitats. (B) In some instances, NH_3_ may concentrate within brine channels or pockets due to exclusion from the ice lattice. (C) Exsolution may occur at regions where liquid water is open to the vacuum or air, such as plume vent regions. (D) These regions may permit volatilization if fractures are open to the surface, leading to either dissolution of NH_3_ into intersecting brine channels higher in the ice shelf or (E) release to the surface. (F) NH_3_ adsorbed onto the ice shell may migrate to brine channels and pockets. (G) Alternatively, NH_3_ could be deposited directly onto and within the ice shell if brine networks are exposed to plumes at the plume vent region.

When considering the permeation of NH_3_ into ice shell niches, we can speculate that several scenarios are possible (Fig. 7). Brine compositions within ice shells could reflect the composition of the ocean from which the brine is derived. Brine channels, veins, pockets, and fractures within the ice could therefore contain NH_3_. Exclusion from the ice lattice during ocean water freezing may even concentrate NH_3_ into the brines (Hammond et al. 2018), and pressure from the ice layer may encourage the formation of ammonia hydrates (Hogenboom et al. 1997, Muñoz-Iglesias and Prieto-Ballesteros 2021). Upward shift of brines into the ice shell may promote exsolution of NH_3_ (i.e., separation of NH_3_ from the liquid phase) and release as a gas. This occurs as per Henry’s Law that states as pressure decreases, as does solubility of gas. However, it should be noted that Henry’s Law is assumed under constant temperature, and temperature would likely change in icy shells.

Fractures that are open to the surface may also enable NH_3_ volatilization, depending on local pH, pressure, and temperature. Volatilization is a process in which a substance transitions from the liquid to the gas phase, escaping into the surrounding air or vacuum. During this phenomenon, the gaseous substance physically leaves liquid and enters the atmosphere or space. Volatilized NH_3_ could migrate along the fracture system and potentially enter other intersecting brine networks within the ice shell. In icy-moon fracture and plume conduits, however, phase partitioning is likely coupled to freezing, boiling, and condensation on cold walls, so NH_3_ transport may occur through a combination of gas flow, adsorption to ice, and re-dissolution into intersecting brines rather than simple free volatilization.

NH_3_ has been shown to adsorb onto ice between −50°C and −25°C (Richter et al. 2025). Plume activity at sites like the tiger stripes of Enceladus could feasibly deliver NH_3_ gas to ice walls. At the ocean-plume interface, brine rises from the ocean into the vent system, where exsolution of NH_3_ occurs. Within the plume shaft, NH_3_ gas could begin condensing onto the ice walls or become incorporated into the ice shell via plume fallback. A hypothetical scenario might proceed as follows: NH_3_ adsorbs onto ice; the adsorbed NH_3_ migrates slowly through the ice shell by surface or bulk diffusion; NH_3_ may enter liquid inclusions within the shell, where it dissolves into liquid brine.

The dispersal and accumulation of NH_3_ in brine channels, veins, pockets, or fractures of icy moons is analogous to NH_3_ volatilization on Earth. NH_3_ losses from ammonium fertilizer application range between 20%–40%, but could be as high as 66%, in pH$\ > \ $7 soils (Powlson and Dawson 2022). NH_3_ has shown to mobilize from the initial, local site of release to distant environments (Sutton et al. 1998, Bouet et al. 2005, Leytem et al. 2024, Lô et al. 2025). Results from the growth of an extremophile, *H. meridiana*, in proximity to an NH_3_ source indicate that NH_3_ from icy moon oceans could alter the potential for habitability not just locally within the ocean, but also at a distance in ice shell brine networks where dispersal of NH_3_ gas is plausible (Hopton and Cockell 2025). Specifically, it was shown that lower concentrations of NH_3_/NH_4_^+^ (i.e., ≤ 0.1 M) improved the growth of *H. meridiana* when cultivated adjacent to the NH_3_ source, possibly by acting as a nitrogen source. Conversely, cultivation of *H. meridiana* proximal to higher concentrations of NH_3_/NH_4_^+^ (i.e., ≥ 0.5 M) significantly reduced growth and viability. The scale of disruption to growth and viability was both distance-dependent and concentration-dependent; cultures in closer proximity to the NH_3_ source showed greater detrimental growth effects, and higher concentrations elicited impacts on growth over a wider spatial range. In the context of icy shells, concentration gradients may arise from a combination of freeze-concentration, fracture transport, and phase partitioning rather than volatilization alone, but nonetheless this factor could influence the propagation of life in a spatial manner.

Given the concentration thresholds for life established earlier, these results indicate the habitability of ice shell environments overlying an ocean of ≈ 0.0181 M NH_3_ on Enceladus may not be limited by the dispersion of NH_3_ into the brine network and could even possibly be enhanced by additional nitrogen input. Conversely, ice shell networks on Titan may be less likely to be habitable when in proximity to the more concentrated ammoniacal ocean. While brine networks higher in the ice shelf could receive little NH_3_ dispersed from the ocean, these networks may also receive fewer nutrients to promote growth. Although the exact NH_3_ concentrations in icy moon oceans have yet to be determined, the foregoing analysis highlights the need to measure the presence and concentration of NH_3_ not just in these oceans, but also within the overlying ice shells, where possibly habitable, and more accessible, niches may exist.

## Conclusions, challenges, and perspectives

The purpose of this review was to present a thorough synopsis of NH_3_ within Enceladus and Titan and infer habitability prospects based upon known microbe-ammonia interactions on Earth. Direct analysis of the plumes of Enceladus indicate NH_3_ is a constituent of the ocean, and models speculate NH_3_ may also be a significant component of the putative ocean of Titan. Both NH_3_ and NH_4_^+^ are essential ingredients for life. NH_4_^+^ is the preferred nitrogen source for many living organisms and forms a vital part of amino acid and protein synthesis, as well as the global nitrogen cycle. However, elevated concentrations of NH_3_ can perturb internal biochemistry and may act to influence habitability prospects.

Bacteria were chosen as biotic models in this review for assessing habitability as a function of NH_3_. Bacteria can survive in extreme conditions but also feature an array of metabolisms that could be suitable for surviving in cold and saline fluids where there is a variety of nutrients and organics. Literature analysis reveals that the highest threshold for growth of a bacteria in NH_3_ extends to 0.5 M, with many bacteria showing survival limits around 0.05 to 0.3 M NH_3_. Research has also demonstrated that dispersion of NH_3_ gas has little detrimental effect on bacterial growth and survival at ≤0.1 M NH_3_. The ocean of Enceladus is estimated to contain ≈ 0.0181 M NH_3_. The comparison of these two data indicates that the survival thresholds of Earth bacteria lie within the speculated concentrations of total ammonia on Enceladus, and thus NH_3_/NH_4_^+^ in the ocean of Enceladus may not limit the potential for habitability. It is also possible that the habitability of overlying ice shell environments may not be limited by the infiltration or dispersion of oceanic NH_3_ into the ice brine networks, if NH_3_ concentrations delivered are similar or lower than those in the ocean.

Conversely, significant NH_3_ abundance (i.e., 0.96 M–9.6 M NH_3_) may constrain the prospects for habitability on Titan. The known survival limits of terrestrial bacteria are below the speculated concentration of NH_3_ in the ocean of Titan. It is possible strains such as *B. pumilus* and *B. pasteurii* could grow in concentrations beyond 0.5 M NH_3_, but this has yet to be demonstrated experimentally. NH_3_ dispersed from a concentrated source at ≥ 0.5 M NH_3_ has also been found to significantly reduce bacterial growth. These comparisons suggest NH_3_ may constrain the potential for habitability of both the ocean of Titan and its overlying ice shell. However, the existence of a global subsurface ocean on Titan remains uncertain, and recent models favour cold, high-pressure ice or localized slushy layers that may be inhospitable (Petricca et al. 2025). The NH_3_-based habitability considerations discussed here are therefore only applicable if liquid or partially liquid ammonia–water phases occur within Titan’s interior.

The comparisons of this review highlight the value of experimentally constraining microbial tolerance to single extraterrestrial parameters. Such data are essential for refining our understanding of biochemical limits to life and for guiding future astrobiological exploration of icy ocean worlds. Yet there are several challenges that prevent more nuanced perspectives. Firstly, we cannot directly compare icy moon oceans to any known environment on Earth. There are terrestrial environments that contain high concentrations of NH_3_ effluent. However, these environments do not present the other physicochemical extremes (e.g., pH, temperature, pressure) that would make them suitable as an analogue for icy moon ocean environments. Moreover, we do not yet have precise physicochemical information regarding the oceans. Icy moon oceans are sealed below ice shells typically tens to hundreds of kilometres thick (Billings and Kattenhorn 2005, Nimmo and Bills 2010, Baland et al. 2014, Čadek et al. 2016, Lucchetti et al. 2017, Levin et al. 2026). While missions could incorporate ice drilling instrumentation, extreme surface radiation and the increasing hardness of ice on icy moons pose significant challenges. Additionally, technological advancements in drilling are limited on Earth as ice sheets reach a maximum thickness of 4.9 kilometres (Fretwell et al. 2013). Due to this, the presence and abundances of salts, volatile organics, and the temperature, pH, and pressure of the oceans can only be estimated from surface observations. In turn, conditions within *in vitro* experiments can only be based upon these estimations or speculative assumptions. This is a particular challenge when considering NH_3_ as a habitability factor: What is the concentration of NH_3_ within the oceans? What species, NH_3_ or NH_4_^+^, predominates in the oceans? Could the pressure and temperature conditions in the oceans sequester NH_3_ into non-toxic solid hydrates? These questions cannot be defined with precision, yet they define toxicity.

It is not without mention that, while this review evaluates habitability using terrestrial biology as a reference framework, another question is this: Could NH_3_ serve as a biological solvent for life, thus rendering its toxic effects irrelevant? Alternative biochemical systems cannot be excluded. Ammoniacal waters have been proposed as potential solvents for hypothetical non-canonical life, and NH_3_-rich environments could in principle support chemistries distinct from those on Earth (Schulze-Makuch and Irwin 2018). Such possibilities fall outside the scope of the present review but underscore that NH_3_ may influence habitability in ways not captured by terrestrial tolerance limits alone.

Although this review has focused on Enceladus and Titan, NH_3_-bearing aqueous environments may occur across a broader range of solar system bodies, and indeed the universe. Localized brine reservoirs are inferred within Ceres (Raymond et al. 2020, Nathues et al. 2022), and subsurface oceans or brine layers have been proposed for Triton (Gaeman et al. 2012), Pluto (Robuchon and Nimmo 2011, Nimmo et al. 2016), Charon (Brown and Calvin 2000, Cheng et al. 2014, Rhoden et al. 2015), the large moons of Uranus (Cochrane et al. 2021, Castillo‐Rogez et al. 2023), and other Kuiper Belt Objects (Hussmann et al. 2006, Brown 2012). These environments extend the relevance NH_3_-derived habitability beyond Saturnian moons. They additionally widen the relevance of any planetary protection implications. Elevated NH_3_ concentrations may inhibit survival or proliferation of many terrestrial micro-organisms, potentially reducing contamination risk in some environments (Deal et al. 1975). Conversely, NH_3_-tolerant or spore-forming microbes carried by spacecraft could persist in local niches comprised of NH_3_ (Kelly et al. 2012). The consideration of NH_3_ is therefore broadly important across icy and volatile-rich worlds for understanding habitability potential and planetary protection. As exploration of icy moons and other extraterrestrial ocean worlds advances, determining the abundance, speciation, and distribution of NH_3_ will be central to assessing both the potential for life and the environments in which life might persist. In this sense, NH_3_ emerges not just as a chemical constituent of these worlds, but as a parameter shaping their habitability.
